# Organoid based personalized medicine: from bench to bedside

**DOI:** 10.1186/s13619-020-00059-z

**Published:** 2020-11-02

**Authors:** Yaqi Li, Peiyuan Tang, Sanjun Cai, Junjie Peng, Guoqiang Hua

**Affiliations:** 1 Department of Colorectal Surgery, Fudan University Shanghai Cancer Center, Shanghai, 200032 China; 2 Department of Oncology, Shanghai Medical College, Fudan University, Shanghai, 200032 China; 3 Institute of Radiation Medicine, Fudan University Shanghai Cancer Center, Shanghai, 200032 China; 4 Cancer institute, Fudan University Shanghai Cancer Center, Shanghai, 230032 China

**Keywords:** Organoids, Stem cells, Disease modeling, Biobanks, Personalized medicine

## Abstract

Three-dimensional cultured organoids have become a powerful *in vitro* research tool that preserves genetic, phenotypic and behavioral trait of *in vivo* organs, which can be established from both pluripotent stem cells and adult stem cells. Organoids derived from adult stem cells can be established directly from diseased epithelium and matched normal tissues, and organoids can also be genetically manipulated by CRISPR-Cas9 technology. Applications of organoids in basic research involve the modeling of human development and diseases, including genetic, infectious and malignant diseases. Importantly, accumulating evidence suggests that biobanks of patient-derived organoids for many cancers and cystic fibrosis have great value for drug development and personalized medicine. In addition, organoids hold promise for regenerative medicine. In the present review, we discuss the applications of organoids in the basic and translational research.

## Background

Two-dimensional (2D) cultured cell lines have been the main *in vitro* research tool for the past decades. Cell lines are relatively cheap, easy to handle and can be applied to multiple experimental techniques. However, the establishment of a cell line is time-consuming and involves extensive genetic and phenotypic adaption to culture conditions. Thus, most cell lines are derived from tumors or have acquired oncogenic potential *in vitro*, while matching normal cells are usually lacking. The main problem of cell lines is the homogeneity of the cells, short of differentiated cell types in the original tissue. These problems limit the use of cell lines in personalized medicine and make them less suited to tissue physiology research requiring differentiated cell types. In cancer research, the preclinical research model that can phenocopy tumor heterogeneity is highly needed for research on the mechanisms of cancer progression and acquired drug resistance. In 1953, the first patient-derived xenograft (PDX) models were successfully established (Toolan 1953). In this model, primary tumor tissue is transplanted into immune-deficient mice, while tumor structure and the relative proportion of tumor cells and stromal cells are largely preserved (Byrne et al. 2017). Thus, PDXs better retain the complexity and heterogeneity of the parental tumor than do cell lines, but establishment is still inefficient and early tumors are hard to establish (John et al. 2011). Besides, genetic manipulations cannot be carried out, and high-throughput analyses are expensive and hampered by complex logistics.

**Fig. 1:**
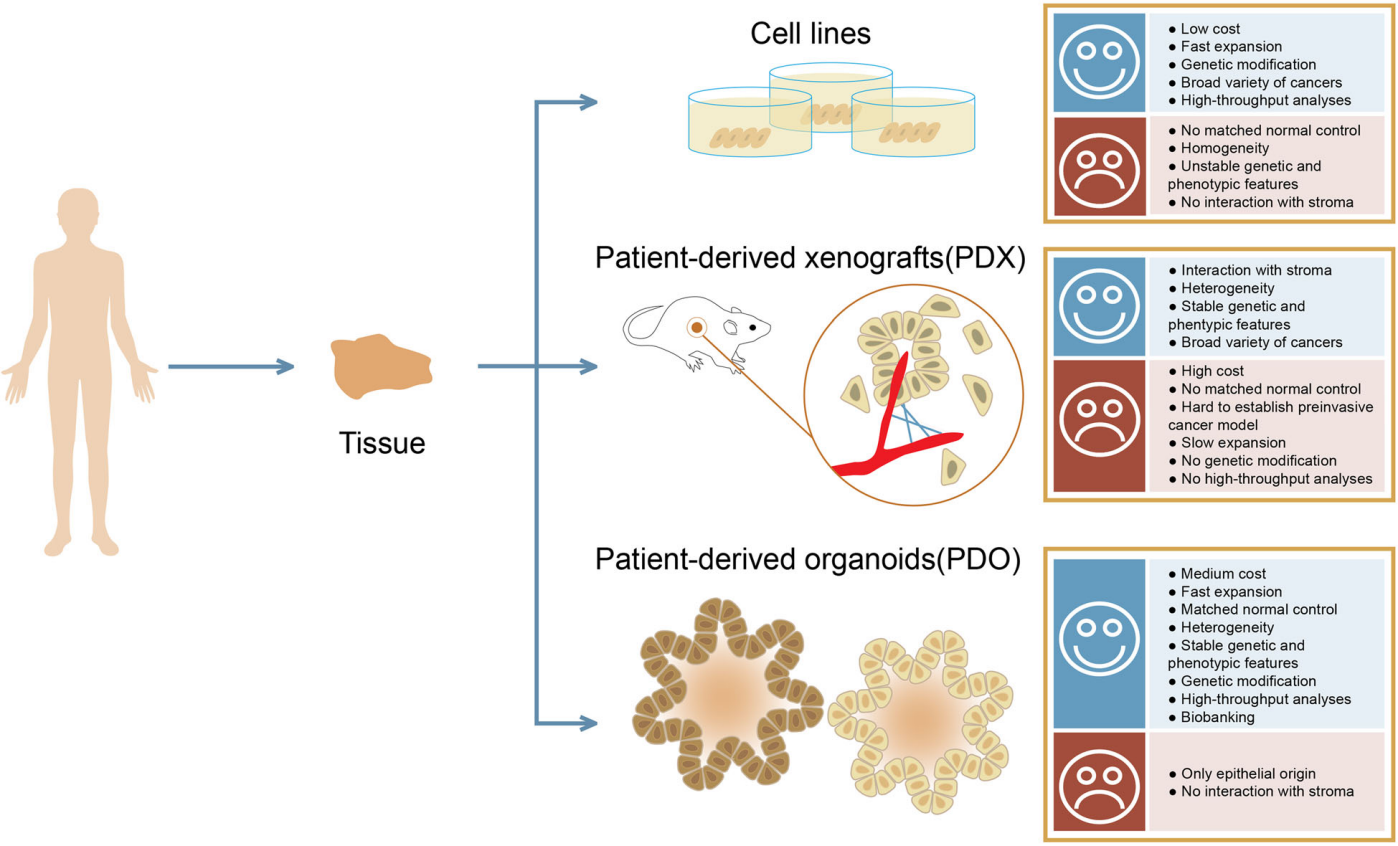
Comparison of cell lines, patient-derived xenografts and organoids. Cell lines have low cost, are easy to handle and can be applied to multiple experimental techniques. PDXs preserve tumor heterogeneity and tumor-stromal interactions. PDOs can be derived from both epithelial cancer cells and normal epithelium and cultured in an extracellular matrix (ECM) -providing basement membrane extract

Last decade has witnessed a booming development of three-dimensional (3D) cell culture technologies. The advent of organoids avoids many of the disadvantages associated with cell lines and PDX. An organoid is characterized as a 3D structure, grown from stem and progenitor cells and consisting of variant organ-specific cell types, that self-organize via cell differentiation and spatially restricted lineage commitment (Clevers 2016). Organoids can be grown from two types of cells: (i) pluripotent stem cells (PSCs), such as embryonic stem cells (ESCs) and induced pluripotent stem cells (iPSCs), or (ii) adult stem cells (ASCs) (Clevers 2016;Rookmaaker et al. 2015). Organoids are proved amenable to all standard laboratory techniques, as well as to genetic modification (Drost et al. 2017;Drost et al. 2015;Schwank et al. 2013). Organoids can be fast expanded, cryopreserved and applied to high-throughput analyses. Though organoid cultures cannot mimic interactions with vasculature and stroma, organoids are a promising research model bridging the gap between cell lines and PDXs (Fig. 1) (Drost 2018; Sachs and Clevers 2014).

### PSCs-derived organoids

Since cell lines of ESCs and iPSCs were established, researchers began to apply insights to induce these stem cells to generate differentiated cell types (Chen et al. 2014; Cherry 2012). Yoshiki Sasai and colleagues firstly dug deeper by questioning whether such an *in vitro* model could mimic *in vivo* development and thus developed methods to culture brain, retina and pituitary structures ‘in a dish’ (Eiraku 2012; Eiraku et al. 2008). Later, iPSCs-derived organoids from optic cup, intestine, stomach, liver, lung, thyroid and kidney, were followed (Chen et al. 2017;Kurmann et al. 2015;McCracken et al. 2014;McCracken et al. 2011;Nakano et al. 2012;Takasato et al. 2015;Takebe et al. 2013). Of note, each germ layer (endoderm, mesoderm, and ectoderm) is represented among this set of organs.

**Fig. 2:**
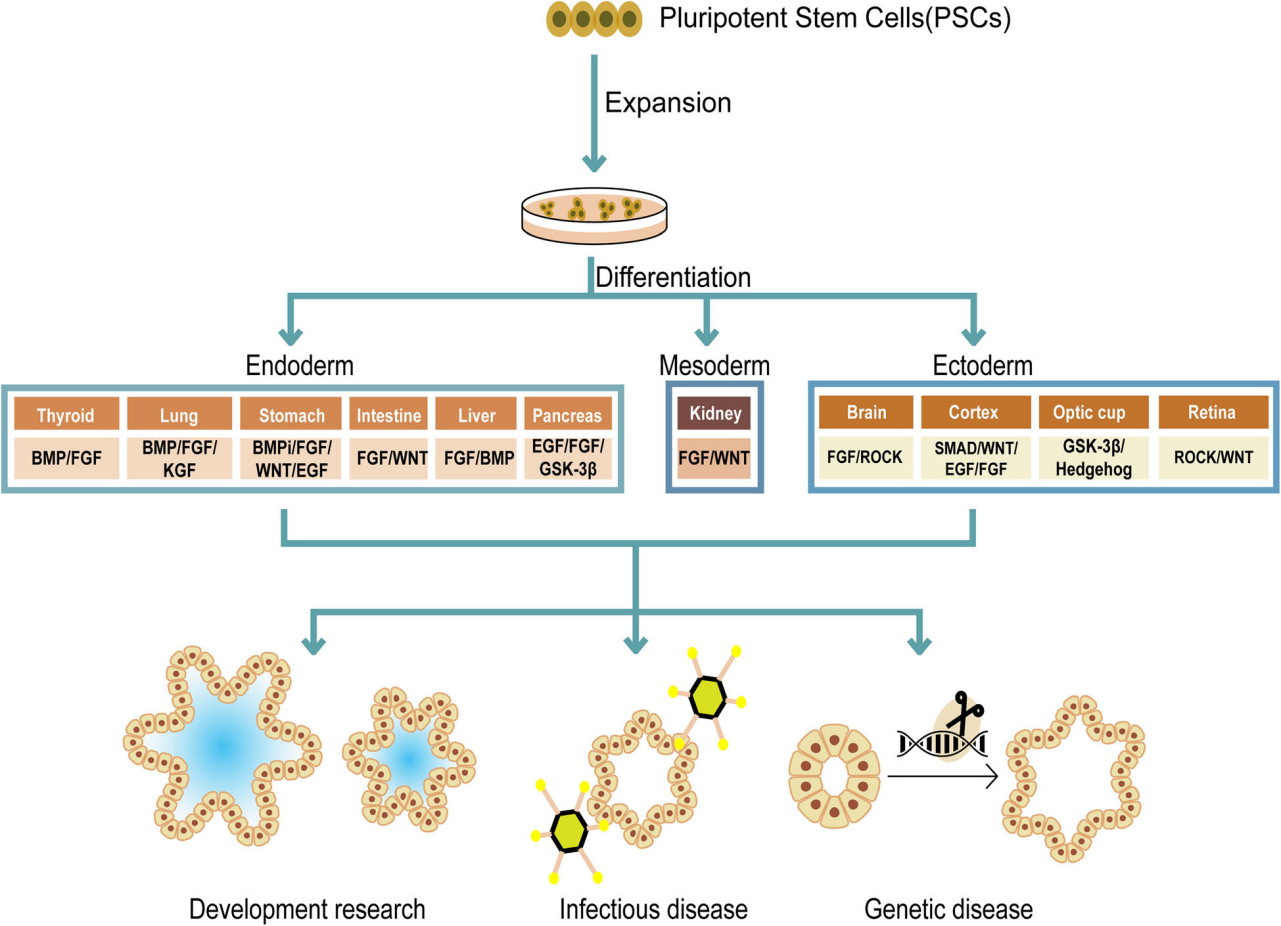
Culture strategy and applications of pluripotent stem cell (PSC)-derived organoids. PSCs-derived organoids can be differentiated toward each of the three germ layers (endoderm, mesoderm, and ectoderm) under specific differentiation signals. PSCs-derived organoids can be applied to studying development, genetic diseases, and infectious disease

Typically, iPSCs are expanded and subsequently differentiated through a multi-step protocol that moves towards a fully differentiated structure, and specific cocktails of growth factors are required for each step (Fig. 2). The differentiation process usually takes about 2-3 months, which depends on the specific type of organ (McCracken et al. 2011). The structure of iPSCs-derived organoids is complex and may contain mesenchymal, as well as epithelial and endothelial components. Because differentiation protocols recapitulate development *in vitro*, iPSCs-derived organoids are excellent models for studying development (Takasato et al. 2015), genetic diseases (Freedman et al. 2015), and infectious disease (Garcez et al. 2016).

**Fig. 3:**
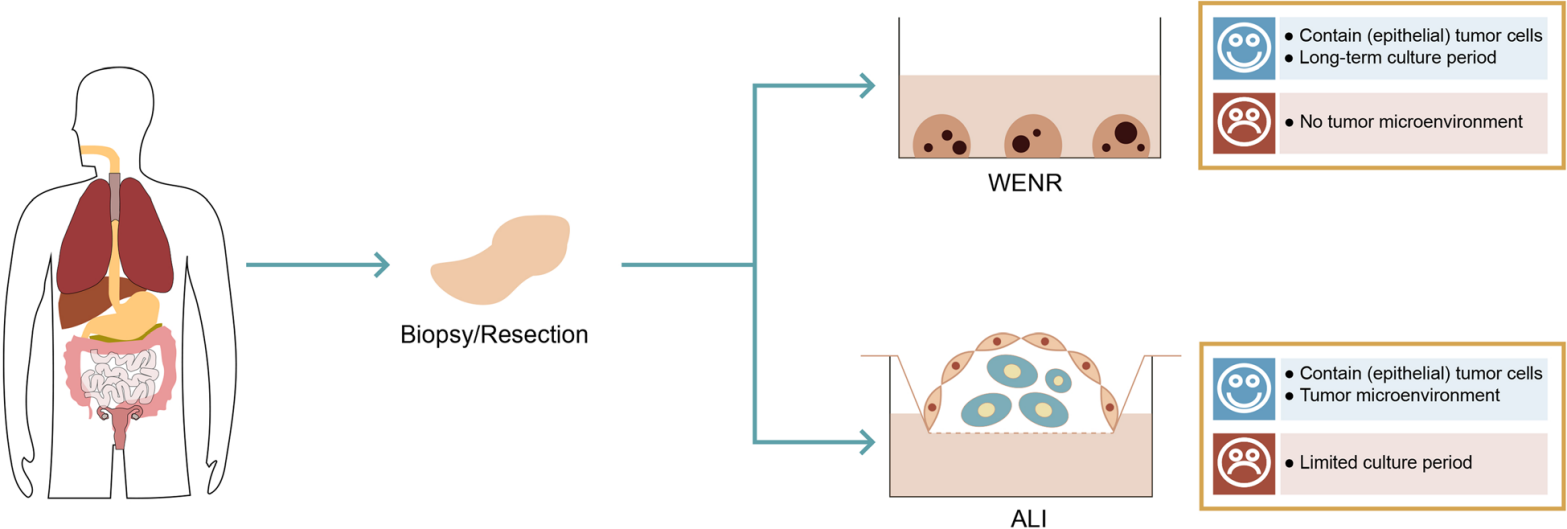
Comparison of different culture methods for adult stem cells-derived organoids. In the WENR method, epithelial organoids are derived from tumor biopsies directly in Matrigel with cocktail growth factors, with long-term expansion but no tumor micro environment. In the air-liquid interphase (ALI) method, tumor biopsies are cultured in ALI in the entire tumor microenvironment as a cell suspension of all cell types, including immune cells and other non-epithelial cell types, but with limited expansion

Another air-liquid interface (ALI) method was introduced allowing for the preservation of both epithelium and matched in vitro stromal microenvironment (Neal et al. 2018). The ALI method employs a Boyden chamber-like structure where primary tissue is seeded in ECM (extracellular matrix) gel in an inner Transwell^TM^ dish which is exposed to air to enhance oxygenation (DiMarco et al. 2014;Li et al. 2014;Ootani et al. 2009). Culture medium is added to the outer dish and can diffuse through the permeable Transwell^TM^ into the inner dish (Fig. 3). ALI method has been applied in PSCs-derived organoid culture lately. Koike and colleagues (Koike et al. 2019) reported the continuous patterning and dynamic morphogenesis of hepatic, biliary and pancreatic structures, invaginating from ALI culture of anterior and posterior gut spheroids differentiated from human PSC. Adapted ALI culture of human cerebral organoids (Giandomenico et al. 2019) and neocortical organoid (Qian et al. 2020) derived from PSCs were also developed.

### ASCs-derived organoids

Complementary to PSCs-derived organoids that recapitulate development *in vitro*, ASCs-derived organoids model adult tissue repair (Clevers 2016) and can be established only from regenerative tissue compartments. In 1987, researches began to explore 3D-culture by culturing primary cells on a reconstituted basement membrane from Engelbreth-Holm-Swarm (EHS) tumor (Li et al. 1987;Shannon et al. 1987). Li and colleagues found mammary epithelial cells cultured on EHS matrix could form ducts, ductules, and lumina and resemble secretory alveoli. Shannon and colleagues cultured adult rat type II cells on EHS matrix with feeder layer cells (mainly fibroblasts) and revealed that cell-matrix interactions help type II cells preserve their original cubical shape and morphological characteristics of variable differentiated cells. Exploration based on ASCs-derived 3D culture has been led to a new stage.

**Fig. 4:**
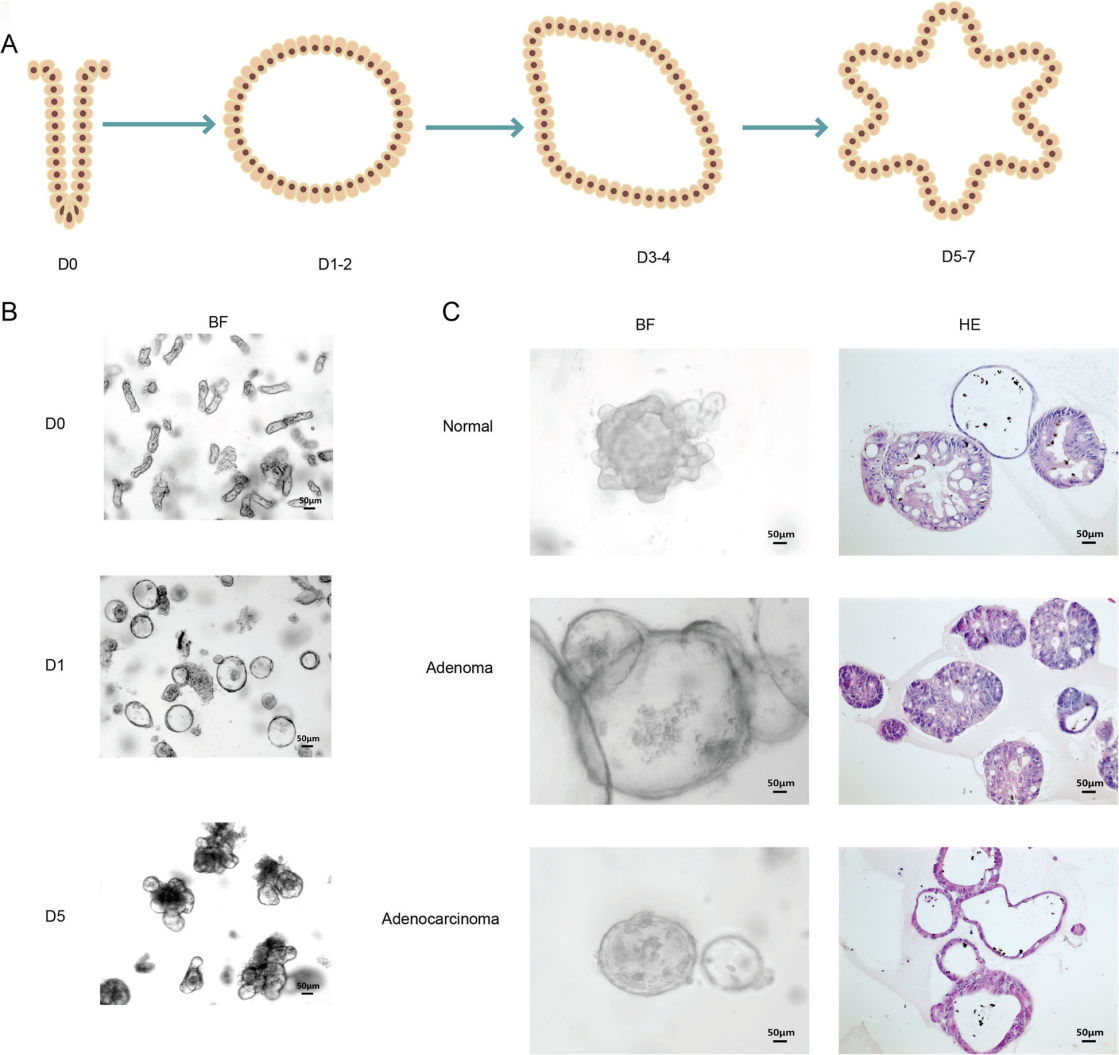
Morphology of several types of human adult stem-cell organoids. **a** A schematic diagram showing the growth pattern of organoids. Typically, isolated cells or functional aggregates are embedded in extracellular matrix domes and cultured in media with essential niche factors. They gradually build tissue-like 3D structures within 1-2 weeks. **b** Bright-field image of a typical murine small intestinal organoid culture. **c** Bright-field image and HE staining image of typical human normal colon epithelium, adenoma and adenocarcinoma organoids

**Fig. 5:**
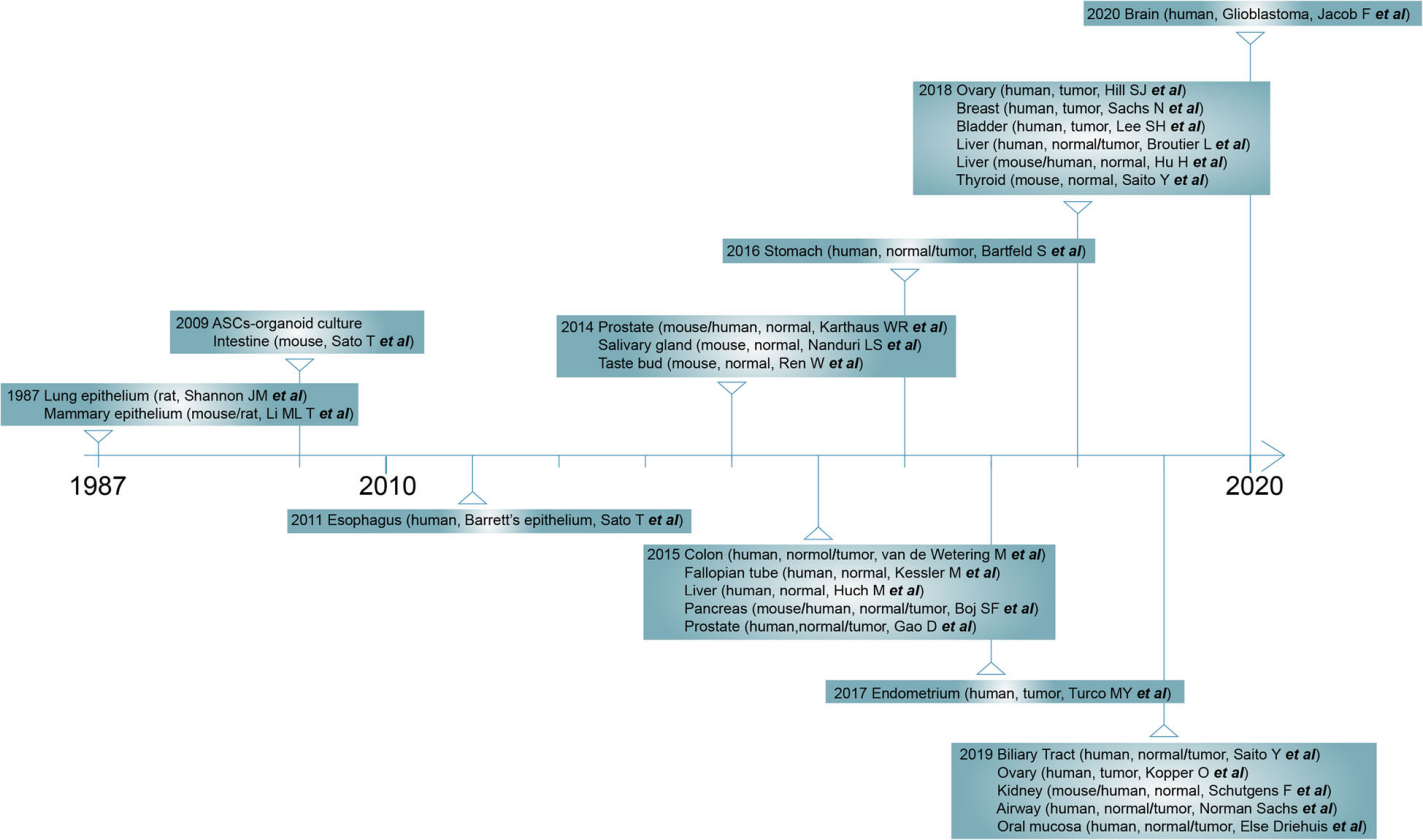
Timeline of adult stem cells (ASCs)-derived organoids development

Two decades later, ASCs-derived organoids were successfully developed from Lgr5-positive intestinal stem cells in culture conditions modeling the stem cell niche of intestine (Sato et al. 2011;Sato et al. 2009). By providing the Wnt agonist R-spondin, epidermal growth factor (EGF), and the bone morphogenetic protein (BMP) inhibitor Noggin, and embedding the cells in an extracellular matrix (ECM) -providing basement membrane extract (WENR method, Wnt3a+EGF+Noggin+R-spondin-1), Lgr5-positive stem cells are able to self-organize, proliferate and form differentiated crypt-villus-like organoids (Fig. 4a, b). Since then, by modifying cocktails of growth factors and cell isolation procedures, cultures of patient-derived organoids (PDOs) have been successfully established for various human tissues by biopsy or resection, including the esophagus (Sato et al. 2011), stomach (Bartfeld et al. 2015), colon (van de Wetering et al. 2015), liver (Broutier et al. 2017;Hu et al. 2018;Huch et al. 2015), pancreas (Boj et al. 2015), salivary gland (Nanduri et al. 2014), fallopian tube (Kessler et al. 2015), ovary (Hill et al. 2018;Kopper et al. 2019), prostate (Gao et al. 2014;Karthaus et al. 2014), breast (Sachs et al. 2018), airway (Sachs et al. 2019), taste buds (Ren et al. 2014), endometrium (Turco et al. 2017), kidney (Schutgens et al. 2019), bladder (Lee et al. 2018), thyroid (Saito et al. 2018), biliary tract (Saito et al. 2019), oral mucosa (Driehuis et al. 2019a) and glioblastoma (Jacob et al. 2020) (Fig. 5, Table 1).

**Table 1:** Comparison of the conditioned media requirements for the patient-derived organoids culture of respective cancer types

brain	head and neck	thyroid	breast	airway	ovary	prostate	bladder	kidney	esophagus	stomach	liver	pancreas	colon
B27	B27	B27	B27	B27	B27	B27	—	B27	B27	B27^a^	B27	B27	B27
—	NACE	NACE	NACE	NACE	NACE	NACE	—	NACE	NACE	NACE	NACE	NACE	NACE
—	NICO	NICO	NICO	NICO	NICO	NICO	—	NICO	NICO	—	NICO	NICO	NICO
—	EGF	EGF	EGF	EGF	EGF	EGF	EGF	EGF	EGF	EGF	EGF	EGF	EGF
—	RSPO	RSPO	RSPO	—	RSPO	RSPO	—	—	RSPO	RSPO	RSPO	RSPO	RSPO
—	Noggin	Noggin	Noggin	—	Noggin	Noggin	—	—	Noggin	Noggin	—	Noggin	Noggin
—	A83-01	A83-01	A83-01	A83-01	A83-01	A83-01	—	A83-01	A83-01	A83-01	A83-01	A83-01	A83-01
—	FGF10	—	FGF10	—	FGF10	FGF10	—	—	FGF10	FGF10	FGF10	FGF10	—
—	FGF2	gastrin	—	—	FGF2	FGF2	—	—	gastrin	gastrin	gastrin	gastrin	gastrin
—	—	SB202190	SB202190	—	SB202190	SB202190	—	—	SB202190	—	HGF	—	SB202190
N2	Forskolin	—	Neuregulin 1	—	—	DHT	DHT	—	N2	—	N2	—	N2
human insulin	PGE2	—	Y27632	—	PGE2	Y27632	Y27632	—	—	—	forskolin	PGE2	PGE2
2-mercaptoethanol	CHIR99021	Wnt3a	—	—	—	—	—	—	Wnt3a	Wnt3a	Wnt3a	Wnt3a	—
(Jaffe et al. 2019)	(Driehuis et al. 2019b)	(Neal et al. 2018)	(Sachs et al. 2018)	(Sachs et al. 2019)	(Hill et al. 2018)	(Chua et al. 2014)	(Lee et al. 2018)	(Schutgens et al. 2019)	(Sato et al. 2011)	(Yan et al. 2018)	(Nuciforo et al. 2018)	(Driehuis et al. 2019c)	(Sato et al. 2011)

^a^B27 without vitamin A is needed for culture of stomach organoid

*Abbreviation*: *NACE* N-acetylcysteine, *NICO* Nicotinamide, *RSPO* R-spondin-1, *PGE2* Prostaglandin E2, *DHT* Dihydrotestosterone

A counterintuitive phenomenon is found that normal epithelium organoids often outgrow tumor organoids, which, in some instances, can be prevented by using cancer-specific selection methods. For example, tumor organoids from colorectal cancer (CRC) can be selectively expanded upon withdrawal of Wnt3a and R-Spondin1. Nearly all CRCs harbor activating mutations in the Wnt pathway or fusion of RSPO(R-spondin-1) genes, allowing for the expansion of cancer cells without Wnts and R-spondins, while normal epithelial cells arrest (Nusse 2017;Sato et al. 2011;Seshagiri et al. 2012;van de Wetering et al. 2015). Another approach to culture tumor cells selectively is to stabilize wild-type P53 by adding the MDM2 inhibitor Nutlin-3 (Drost et al. 2015). Tumor cells are not affected by Nutlin-3 due to a loss of TP53 (Olivier et al. 2010), while normal cells in culture present cell cycle arrest and death, allowing for the selection of tumor cells.

**Fig. 6:**
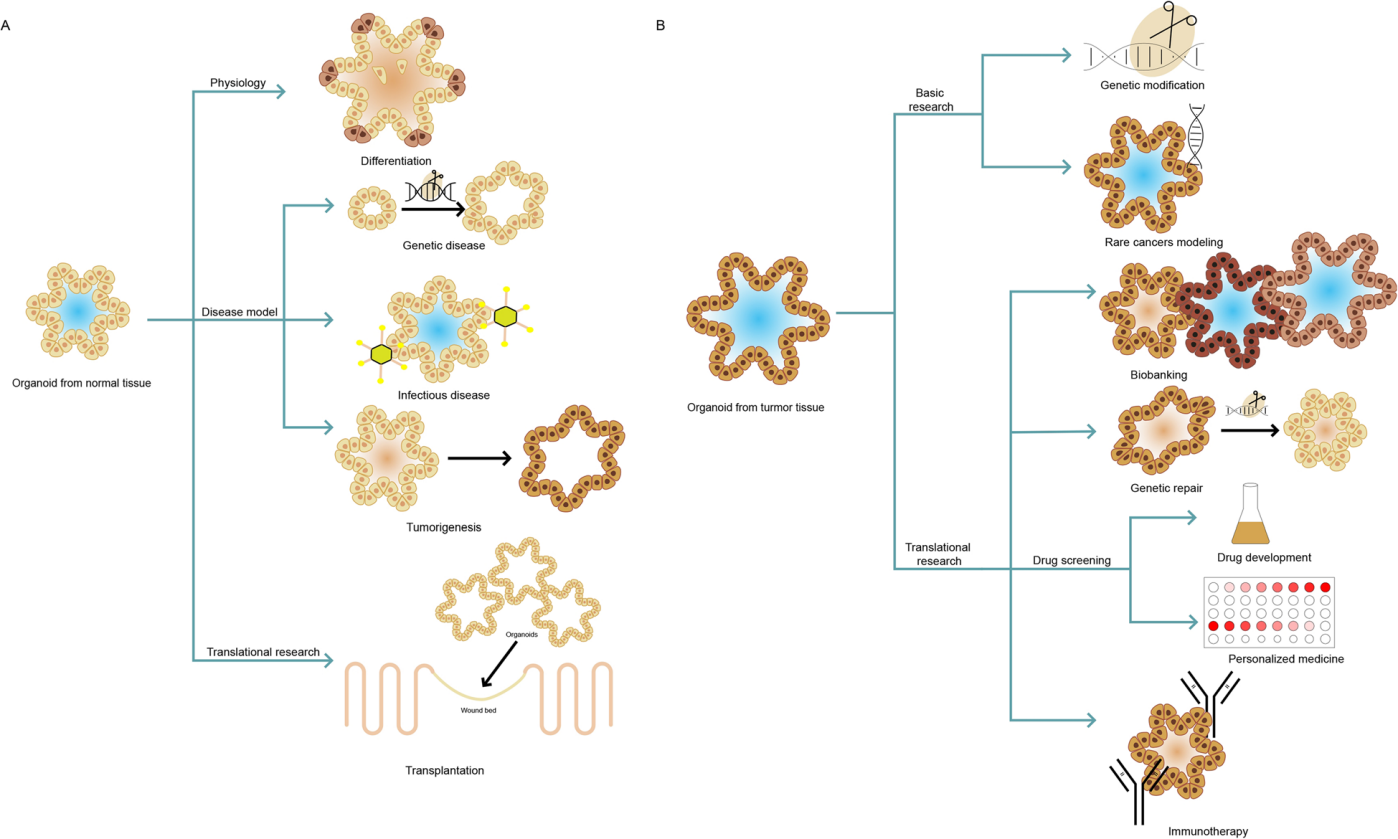
Applications of adult stem cells-derived organoids. **a** Organoids derived from normal tissue are useful for studying physiology. For disease modeling, organoids can be genetically engineered to model genetic and malignant diseases by using CRISPR-Cas9. Normal organoids can also be infected with different types of pathogens to model infectious disease. Normal organoids can be transplanted to wounds for tissue repair. **b** Tumor-derived organoids can be used for basic research by genetic modification and modeling rare cancer. For translational research, tumor-derived organoids can be used for biobanking, genetic repair and drug screening studies, both for personalized medicine (to choose the most effective treatment for a specific patient) and drug development (to test a compound library on a specific set of tumor organoids), as well as immunotherapy research

In general, PDOs using WENR method can be derived from any epithelium of normal tissues as well as malignant or otherwise diseased tissues within approximately 7 days after embedding the cells into ECM matrix (Fig. 3c; Fig. 5). PDOs can be expanded long term and cryopreserved while remaining genetically stable, making organoids an ideal tool for disease modeling. In addition, this type of organoid culture allows the direct parallel expansion of diseased cells and matched normal cells from individual patients, which allows for the generation of living tumor organoid biobank and facilitates its potential application in personalized therapy (Fig. 6). However, to date, nearly all PDOs types represent only the epithelial parts of organs, and there is an absence of stroma, nerves, and vasculature.

Adopting ALI method, researchers can generate ASCs-derived organoids from various murine tissues including small intestine, colon, stomach, and pancreas (Li et al. 2014;Ootani et al. 2009), then extending to culture clinical tumor samples (Neal and Kuo 2016; Neal et al. 2018), accurately recapitulating stem cell populations and their multi-lineage differentiation. The ALI model preserves tumor microenvironment with tumor parenchyma and stroma, including functional tumor infiltrating lymphocytes (TILs), providing a promising model for immunotherapy research for patients with cancer (Neal et al. 2018).

In the remainder of this review, we will discuss how PSCs-derived organoids and ASCs-derived organoids are applied in basic and translational research.

## Organoids for basic research

### Tissue physiology

#### Organoids as a research tool for stem cell biology

Organoids is an ideal *in vitro* tool for the identification of novel stem cell markers, and the study of physiological phenomena requiring the coculture of multiple cell types. Lgr5+ cells located at the crypt base was verified as the real intestinal stem cells (Barker et al. 2007). Enlightened by the finding that Lgr5^+^ intestinal stem cells can undergo thousands of cell divisions *in vivo*, Sato and colleagues (Sato et al. 2011;Sato et al. 2009) successfully established the epithelial organoids from a single Lgr5+ stem cell, which are also known as “mini-guts”. Wnt signals, Notch signals, EGF signals and BMP signals together contribute the stem cell niche homeostasis (Clevers 2013). Other stem cell biomarkers have been explored to initiate intestinal organoid cultures, including CD24 (von Furstenberg et al. 2011), EphB2 (Jung et al. 2011), and CD166+/GRP78 (Wang et al. 2013). Mini-guts contain multiple differentiated cell types. Rapidly dividing, transit-amplifying (TA) daughter cells derived from Lgr5^+^ cells can differentiate to enterocytes, Paneth cells, goblet cells, enteroendocrine cells, tuft cells, and the M cells that cover Peyer’s patches (Clevers 2013),contributing to the study of physiology of crypt-villus axis. Lendeboom and colleagues (Lindeboom et al. 2018) applied a multi-omics framework on stem cell-enriched and enterocytes-enriched mouse intestinal organoids to reveal multiple layers of gene expression regulation contributing to lineage specification and plasticity of intestine and found that Hnf4g as a major driver of enterocyte differentiation. As another example, Beumer and colleagues (Basak et al. 2017;Beumer et al. 2018) used organoids to study the effect of growth factors on hormone expression in enteroendocrine cells after establishing a protocol to obtain enteroendocrine cells in organoids. In organoids, hormones in enteroendocrine cells were differentially expressed based on the presence or absence of BMP4. This finding was then studied in a mouse model, and it was found that the BMP gradient along the crypt-villus axis *in vivo* dictates a switch in expressed hormones in enteroendocrine cells that migrate up this BMP gradient. Beumer and his colleagues (Beumer et al. 2020) further constructed an organoid-based platform for functional studies of human enteroendocrine cells, which can be induced by transient expression of *NEUROG3*. By using single-cell mRNA sequencing and mass-spectrometry, they revealed differences of human enteroendocrine cells with mice, and several secreted products were identified and validated by functional experiments.

The mini-gut culture approach has been applied to the generation of organoids derived from the epithelial compartments of a variety of murine and human tissues of ecto-, meso- and endodermal origin, and promotes the study of stem cell biology of other tissues except for intestine. For example, long-term expanding organoids modeling mature pyloric epithelium can be efficiently generated from single Lgr5^+^ stem cells located at the base of pyloric glands (Barker et al. 2010). Later, Strange and colleagues (Stange et al. 2013) discovered that Troy^+^ chief cells can spontaneously dedifferentiate to act as multipotent epithelial stem cells *in vivo*, particularly upon damage. Importantly, single Troy^+^ chief cells can initiate long-term expanding gastric organoids, containing various cell types of corpus glands. The finding further confirms Troy^+^ chief cells’ role as “reserve” stem cells upon challenge of tissue homeostasis.

#### Organoids for generation of specific cell types

Organoid culture allows for the generation of specific cell types that were previously impossible in 2D cultures. For example, hepatocytes can be successfully established and expanded in organoid culture (Hu et al. 2018;Peng et al. 2018). Based on adult bile duct-derived bipotent progenitor organoids (Huch et al. 2015), culture conditions were developed that supported the growth of human hepatocyte organoids. The organoids proliferate greatly after transplanting into mice (Hu et al. 2018). The resulting hepatocytes maintained its original physiological functions, including secreting cytoplasmic glycogen particles, forming bile canaliculi, and expressing albumin and cytochrome P450 enzymes. Based on organoid culture system of hepatocytes, Peng and colleagues (Peng et al. 2018) described a unique effect of tumor necrosis factor-α, a cytokine essential for liver regeneration and found that the addition of regeneration-enhancing cytokines in facilitating the *in vitro* expansion of cell types that are otherwise difficult to culture. As another example, Yin and colleagues (Yin et al. 2014)showed modulation of Wnt and Notch signaling in intestinal organoids to direct lineage differentiation into mature enterocytes, goblet cells and Paneth cells. Specifically, the combination of IWP-2 (Inhibitor of Wnt Production 2; Wnt pathway inhibitor) and VPA (valproic acid; Notch activator) specifically induced enterocyte differentiation, presumably by combining the effects of both inhibitors, in which IWP-2 induced enterocyte differentiation while VPA suppressed the differentiation of Lgr5+ stem cells toward secretory cell types. The combination of DAPT (Notch inhibitor) and CHIR (chicken immunoglobulin-like receptor; GSK3β inhibitor) mainly induced Paneth cell differentiation, and the combination of IWP-2 and DAPT primarily induced goblet cell differentiation. These methods provide new tools for the study and application of multiple intestinal epithelial cell types.

#### Organoids as a genetically stable in vitro research tool

Organoids can be established from a single cell, which makes it possible to study the mutational status of single stem cells. The gradual accumulation of genetic mutations in stem cells throughout life is related to a variety of age-related diseases, including cancer. In this way, Blokzijl and colleagues (Blokzijl et al. 2016) were able to unveil mutation rates and patterns in normal stem cells throughout life by whole-genome sequencing (WGS) analysis (with peripheral blood as a reference for germline mutations). Interestingly, the mutation rate, with around 40 novel mutations per year per stem cell, was similar in liver, small intestine, and colon stem cells, regardless of the large variation in cancer incidence of these organs. However, the types of mutations detected and the resulting mutational signatures in colon and small intestine cells were different from those in liver cells. To be pointed out, the inter-individual variation in mutation rate and spectra are low, indicating organ-specific activity of common mutational processes throughout life.

### Disease modeling

#### CRISPR-Cas9 technology as a useful tool for disease modeling of organoids

The clustered regularly interspaced short palindromic repeats (CRISPR) associated protein 9 (Cas9)/CRISPR system has become a major technology for mammalian genome editing. The system consists of Cas9 nuclease derived from Streptococcus pyogenes and guide RNA which can recognize and target a specified DNA sequence preceding the motif sequence adjacent to the. CRISPR-Cas9 can generate DNA double-strand breaks at specific genomic sites. Mammalian double-strand DNA breaks can be repaired by two ways, non-homologous end joining (NHEJ) and homology-directed repair (HDR). NHEJ inserts indels randomly in the process of repair, and biallelic introduction of indels leads to gene knock-out (Komor and Badran 2017). On the other hand, HDR can replace the damaged allele by existing intact genome, thus when tailored DNA templates are co-delivered with CRISPR-Cas9, HDR can be used for gene knock-in (Komor and Badran 2017).

**Fig. 7:**
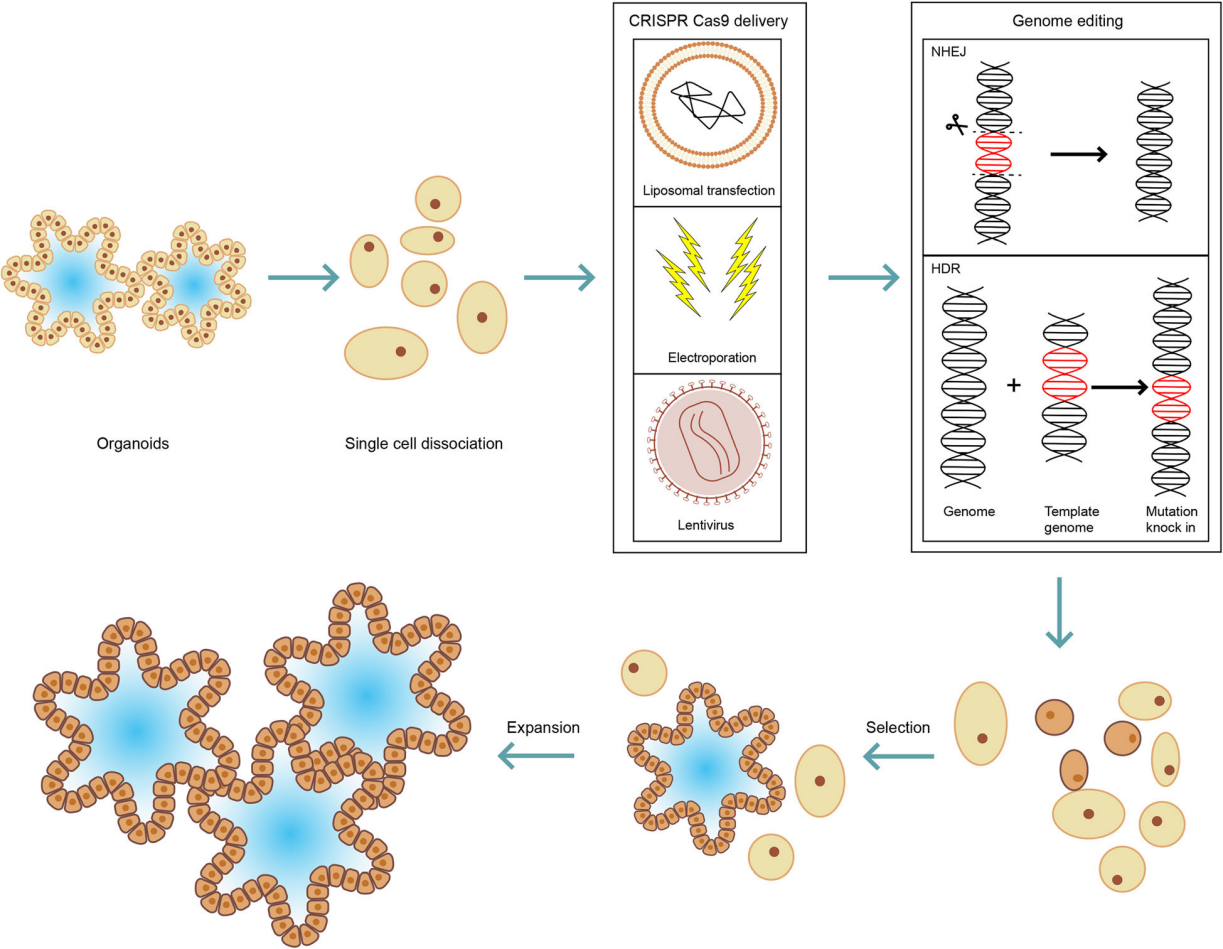
Genome editing in organoids by CRISPR-Cas9 technology. Workflow of genetic engineering in organoids using CRISPR-Cas9. Either NHEJ or HR can be exploited for gene knock-out or knock-in, respectively. In most cases, expansion of single organoid clones after the selection procedure is necessary to obtain isogenic organoid populations

Although the CRISPR-Cas9 technology has broadened its applications to a series of purposes, including DNA base editing, RNA targeting, epigenome editing and gene expression manipulation (Adli 2018;Komor and Badran 2017), the use of CRISPR-Cas9 on organoids still basically harness NHEJ and HDR to engineer genes of interest. Indeed, organoids are ideal model for investigating gene function by genome editing, as the organoid system allows for fast expansion with stable genetics and phenotype. Previous studies have successfully achieved genome editing by installing CRISPR-Cas9 into organoids using various approaches, including liposomal transfection, electroporation and viral infection (Fig. 7). However, variable experimental conditions limit the efficiency of genome editing in organoids, including the recovery after single-cell isolation, approaches for CRISPR-Cas9 delivery, and the cleavage efficiency of the guide RNA. Selection and enrichment of positive organoids are necessary after CRISPR-Cas9-mediated genome editing, or otherwise, labor-intensive organoid cloning, followed by sequencing of expanded organoid clones is needed. Recently, Ringel and his colleagues (Ringel et al. 2020) developed a genome-wide pooled-library CRISPR screen approach by capturing sgRNA (single-guide RNA) integrations in single human intestinal organoids to dissect oncogenic signaling pathways. Their screening method would be broadly applicable to various organoid models and selection assays, which may contribute to dissecting human disease mechanisms and facilitating biological discovery in primary 3D tissue models.

#### Genetic disease

Currently, organoids have become a useful tool to model genetic diseases. Generally, two types of methods have been adopted: (i) organoids established from patient-derived biopsies; (ii) specific genetic mutations introduced to wild-type organoids using CRISPR-Cas9 technology.

#### Cystic fibrosis

Cystic fibrosis (CF) is the best example for PDO modeling human genetic disease. CF is a monogenic channelopathy caused by inactivating mutations in the CF transmembrane conductance regulator (CFTR) gene. The disease involves multiple organs, including the intestine, lung, pancreas, liver, kidney and sweat gland. In gastrointestinal organs and lungs, decreased CFTR function results in reduced chloride transport through CFTR toward the extracellular space, leading to a reduced water flow by osmosis and, hence, increased density of mucus. In the sweat glands, loss of CFTR function leads to a high saline concentration in sweat.

Organoid model was firstly derived from rectum of CF patients. Early work with organoids derived from the rectum of CF patients revealed CFTR function: wild-type organoids rapidly swell upon opening the CFTR channel in a cyclic adenosine monophosphate (cAMP)-dependent manner through the addition of forskolin (FSK) (Dekkers et al. 2013). Rectal organoids from CF patients do not respond to FSK, but it is restored upon pre-incubation with CFTR-restoring compounds (Dekkers et al. 2013) or upon correction of the CFTR mutation by CRISPR-Cas9 (Schwank et al. 2013). Based on this finding, this organoid-based FSK-induced swelling assay began to be used to test drug response on organoids isolated from patients harboring different CFTR mutations, including rare variants (Dekkers et al. 2013).

Lung is another organ that can be severely harmed by CF. Due to CFTR mutation, a thick sticky mucus forms in the lungs, which impairs breathing and provides a fertile environment for pathogens reproduction, leading to premature respiratory failure. CF airway organoids can be established from patient-derived iPSCs (Firth et al. 2015), bronchial epithelial cells based on both ALI (Fulcher et al. 2005) and WENR cultures (Sachs et al. 2019) or bronchoalveolar lavage fluids (no biopsy needed) (Sachs et al. 2019;Sondo et al. 2014). Airway organoids from CF patients had an increased mucus layer, recapitulating the disease phenotype. FSK-induced swelling in airway organoids was reduced compared with organoids from normal controls and could be restored with CFTR-restoring compounds. However, in contrast to rectal organoids, FSK-induced swelling in lung organoids did not depend on CFTR alone, it was also influenced by the chloride transporter TMEM16A, which is set as an alternative therapeutic target for CF patients. Therefore, airway organoids may function as an additional platform for assessing drug response to CF, particularly for drugs acting on TMEM16A.

Besides, liver, pancreatic and kidney organoids derived from CF patients can also be established. Sampaziotis and colleagues (Sampaziotis et al. 2015) generated iPSCs from skin fibroblasts of a CF patient with homozygous F508 mutation and differentiated them into cholangiocyte-like cells (CLC). CF-CLCs expressed displayed defective expression of CFTR protein. CF-CLC organoids treated with experimental CF drug VX809 increases CFTR function and improves intraluminal fluid secretion. CF pancreatic organoids can be established from PSCs of CF patients, including both human ESCs and an iPSC line, to generate differentiated pancreatic ductal epithelial cells (PDECs) (Simsek et al. 2016). PDECs derived from CF-iPSCs showed decreased expression of CFTR protein and damaged chloride ion channel activity, reappearing functional defects of patients with CF at the cellular level. In addition, a tubuloid line from urine of a CF patient was established (Schutgens et al. 2019). At the morphological level, the kidney tubuloids maintained folded over long-term culture, instead of the typical cystic phenotype, which was probably due to the lack of CFTR function caused by CFTR mutations F508del/S1251N. In tubuloids derived from urine of CF patients, FSK caused slight swelling in a concentration-dependent manner, suggesting residual CFTR function, while after pre-incubation with the CFTR-potentiator drug VX-770 (ivacaftor, Kalydeco), swelling increased significantly. All the above CF organoid models of different related organs allow *in vitro* assessment of treatment response and development of novel drugs. For a discussion of the use of CF-PDO for personalized medicine, see Section 3.1.

#### Intestinal genetic diseases

Additionally, intestinal organoids harboring inactivation mutation of *TTC7A* have been successfully derived from patients with intestinal atresia, which recapitulates how *TTCA7* deficiency results in the loss of apical-basal cell polarity in the intestinal epithelium that can be rescued by adding Rho kinase inhibitors (Bigorgne et al. 2014). Besides, intestinal organoids were derived from patients with microvillus inclusion disease (MVIX) caused by homozygous truncating mutations of *syntaxin-3 (STX3)* gene. The model revealed that partial loss of brush border microvilli and subapical accumulation of vesicles are typical histological phenomena of the disease (Wiegerinck et al. 2014).

#### Liver genetic disease

Liver organoids have been generated from patients with α1-antitrypsin (A1AT) deficiency. Accumulation of mutant *A1AT* in the endoplasmic reticulum in the liver leads to fibrosis or cirrhosis. Liver organoids derived from the patients indeed contained A1AT aggregates and presented increased apoptosis, which might contribute to fibrosis and cirrhosis (Huch et al. 2015). Alagille syndrome is caused by loss-of-function mutations in *JAG1* or *NOTCH2* and leads to partial or complete biliary atresia. Accordingly, organoids generated from a patient with Alagille syndrome can not differentiate toward the biliary fate, whereas in proliferate conditions, no differences were observed compared with healthy controls (Andersson et al. 2018;Sachs et al. 2018).

#### Other genetic diseases model based on iPSCs-derived organoids

iPSCs-derived organoids can also be manipulated by CRISPR-Cas9 technology to model diseases in different tissues. In human iPSCs-derived kidney organoids, knock-out of podocalyxin (Kim et al. 2017) and *PKD* genes (Cruz et al. 2017;Freedman et al. 2015) recapitulated defects that mimic nephrotic syndrome and polycystic kidney disease respectively, as well as contributed to understanding the functions of the genes in the pathogenesis context. Engineered iPSCs-derived liver organoids helped in illustrating the various functions that different mutations of *JAG1* gene can have in the development of bile ducts and genesis the Alagille syndrome: the C829X mutation of *JAG1* can causes significant alterations, while the G274D mutation does not affect organoid properties (Guan et al. 2017).

In brain tissue, patient-specific iPSCs-derived brain organoids can be used to model lissencephaly (Bershteyn et al. 2017), Down syndrome (Xu et al. 2019), and neuronal heterotopia(Klaus et al. 2019). Engineered iPSCs-derived brain organoids were established to model microcephaly by RNA interference of reprogramming factors (Lancaster et al. 2013), autism by overexpression of the transcription factor FOXG1 (forkhead box G1) (Mariani et al. 2015), macrocephaly by deletion of PTEN (Li et al. 2017), Timothy syndrome by introducing mutations in the CaV1.2 calcium channel-interneurons (Birey et al. 2017) and Aicardi-Goutières syndrome by introducing inactivation mutation of *TREX-1* (Thomas et al. 2017).

Fused organoids culture was recently established to understand more complex biology, which was more applied in brain study. Bagley and colleagues (Bagley et al. 2017) firstly showed a co-culture method combining brain regions of choice within one organoid tissue and they generate a dorsal-ventral axis by fusing organoids of dorsal and ventral forebrain identities. Combined with reprogramming technology, their novel fusions of organoids culture should offer researchers the possibility to analyze complex neurodevelopmental defects using cells from neurological disease patients and to test potential therapeutic compounds. Xiang and his colleagues (Xiang et al. 2017) successfully established and fused medial ganglionic eminence (MGE) and cortex-specific organoids from human pluripotent stem cells followed by live imaging, to investigate MGE development and human interneuron migration and integration, which offers deeper insight into molecular dynamics during human brain development . The same research team developed a new 3D system to create the reciprocal projections between thalamus and cortex by fusing the two distinct region-specific organoids, providing a platform for understanding human thalamic development and modeling circuit organizations and related disorders in the brain (Xiang et al. 2019) .Generally, engineered organoids can faithfully recapitulate genetic diseases and thus provide a valid resource for basic research and for development of novel therapeutics.

#### Infectious disease

**Fig. 8:**
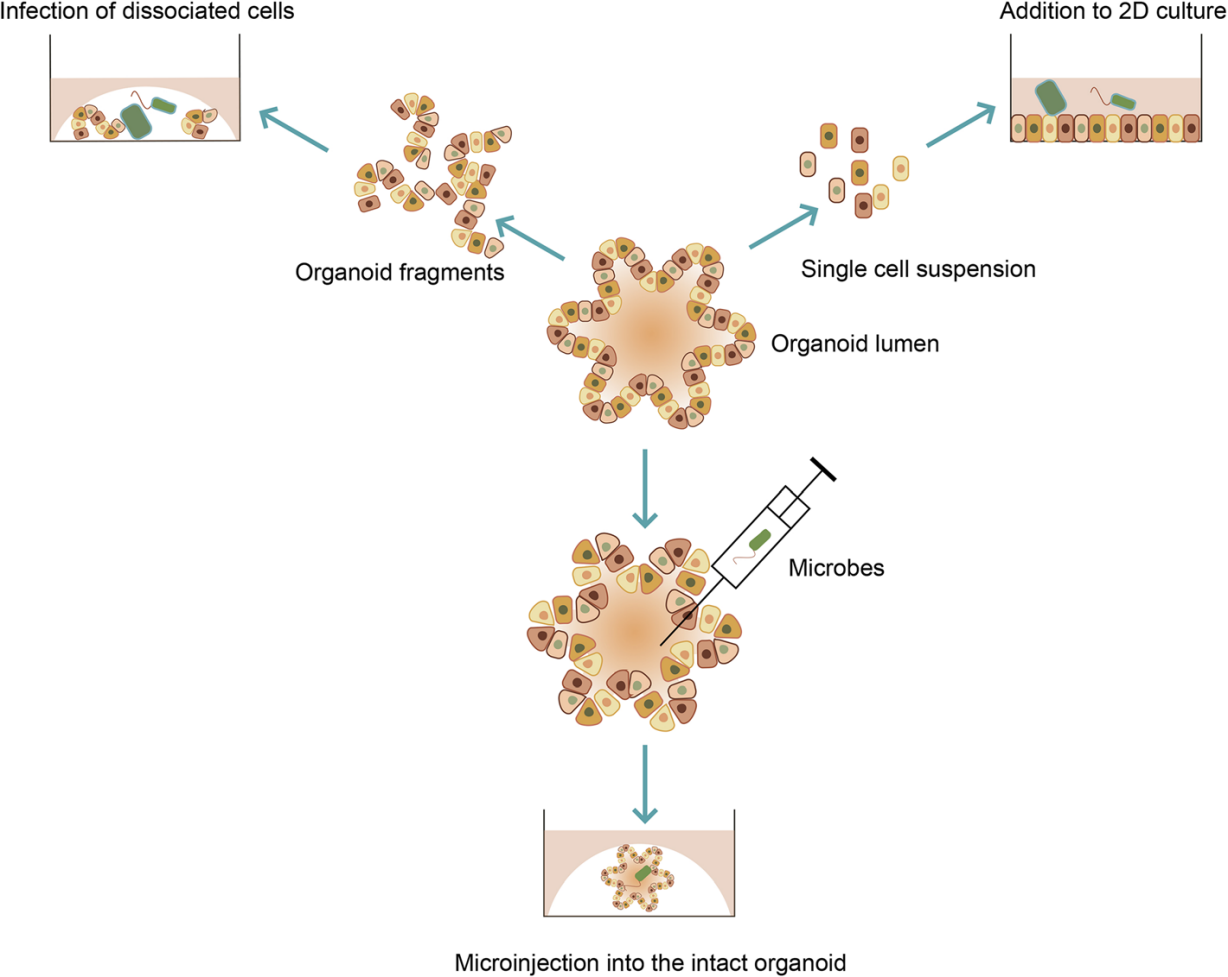
Approaches of studying infectious diseases using organoids. Organoids can be micro-injected with the microbe, thus the microbe is in direct contact with the apical side of epithelial cells, which became the mainstream method to build infection model. Organoids can also be sheared into smaller aggregates, incubated with pathogens and re-seed into Matrigel. Alternatively, 3D organoids can be digested by enzyme into single cells and grown as 2D monolayer cultures, and then microbes are added into the culture media

Organoids are closed 3D structures that exhibit the apical side of the epithelium towards the lumen and the basal membrane towards the outside. The apical membrane within the lumen is initially exposed to pathogens *in vivo*. Three different methods have been established to reproduce the interaction between microbes and the host in the organoids culture (Fig. 8).

In the first method, organoids are mechanically sheared or digested into single-cell suspensions or large particles and then they are incubated with pathogens, which leads to infection of the cells. After embedded in the 3D matrix, infected cells can reform organoids that can be used to model infectious disease (Dang et al. 2016;Forbester et al. 2015;Nigro et al. 2014;Zhang et al. 2014). This method is easy to handle and do not require special equipment. But, the efficiency of infection varies among different pathogens and it can not reflect the initial interaction between microbes and the host. Besides, during the process not only the apical side but also the basal side of cells are exposed, thus nonspecific responses may be introduced due to the interaction of pathogens with the basal side of the cells.

In the second method, pathogens are directly injected in to the lumen of the organoids by a microinjection, thus the initial interaction of pathogens and the early response of the host cells can be captured and either apical or basal interaction can be investigated separately (Bartfeld et al. 2015;Leslie et al. 2015;McCracken et al. 2014). This method is now the mainstream method to build infection model. However, this method needs special such as a microinjector and it is hard to perform quantitatively due to the different sizes of organoids.

In the third method, when single cells digested from organoids are seeded onto a 3D matrix-coated dish, they grow as 2D monolayer with the apical side exposed. Adding pathogens directly into the culture media allows interaction between microbes and the host cells (Ettayebi et al. 2016). The 2D culture contains various differentiated cells and allows quantitative experiments, while it does not resemble the *in vivo* 3D structure of host tissues.

Based on the above approaches, organoids have been adopted to model viral, bacterial, and parasitic infectious diseases of different tissues, including diseases caused by pathogens that previously could not be studied *in vitro* (Table 2). These models recapitulate features of *in vivo* infection and could help identify therapeutic targets and develop novel drugs and vaccine.

**Table 2:** Organoids of different organs being used for studying infectious diseases

Organoid		Brain	Liver	Intestine	Stomach	Gallbladder	Kidney	Respiratory Tract	Head and Neck
Pathogen	Virus	Zika virus	Hepatitis B virus(HBV)	Human Norovirus (HuNoV)	—		BK virus	Respiratory syncytial virus (RSV)	HSV1
		Japanese encephalitis virus	—	Rotavirus	—		—	Influenza virus	HPV
		—	—	SARS-CoV-2	—		—	Enterovirus 71 (EV71)	
	Bacterium	—	—	*Salmonella typhi (S. typhi)*	*Helicobacter pylori (H. pylori)*	*Salmonella*	—	—	
		—	—	*Clostridium difficile (C. difficile)*	—		—	—	
	Parasite	—	*Plasmodium*	*Cryptosporidium*	—		—	*Cryptosporidium*	
Source		PSCs	PSCs & ASCs	PSCs & ASCs	PSCs & ASCs	ASCs	ASCs	PSCs & ASCs	ASCs
Reference		(Cugola et al. 2016;Dang et al. 2016;Gabriel et al. 2017;Garcez et al. 2016;Wells et al. 2016;Xu et al. 2016)	(Chua et al. 2019;Ng et al. 2015;Nie et al. 2018)	(Engevik et al. 2015;Ettayebi et al. 2016;Finkbeiner et al. 2012;Forbester et al. 2015;Heo et al. 2018;Lamers and Beumer 2020;Ramani and Atmar 2014;Yin et al. 2015)	(Bartfeld et al. 2015;Huang et al. 2015a;McCracken et al. 2014;Schlaermann et al. 2016)	(Scanu et al. 2015)	(Schutgens et al. 2019)	(Heo et al. 2018;Hui et al. 2018;Persson et al. 2014;van der Sanden et al. 2018;Zhou et al. 2018)	(Driehuis et al. 2019a)

#### Viral disease

PSCs-derived organoids were firstly used to model viral disease. In neuroscience, the development of PSCs-derived brain organoids help *deciphered the sequence of disease progression in Zika virus*(Garcez et al. 2016;Qian et al. 2016). *Zika virus* (ZIKV) mainly spreads by the Aedes aegypti mosquito and its infection can lead to microcephaly, which was set as a public health emergency by World Health Organization (WHO) in 2016. However, the pathogenesis of the ZIKV infection was not fully understood until brain organoids emerged. Multiple research demonstrated that ZIKV infection can cause disorder of the cortical layers of cerebral organoid, abrogating growth and thus halting neurogenesis. Researchers found that ZIKV infection leads to the activation of the Toll-like receptor 3 (TLR3), contributing to deregulated neurogenesis and decreased functional neurons (Cugola et al. 2016). Gabriel and colleagues (Garcez et al. 2016) further illustrated that two new isolated ZIKVs have different patterns of pathogenicity. Unlike the highly passed MR766 strain of ZIKV, the new strains, infected apical proliferating progenitors, interfering with centrosomal protein assembly, which in turn led to their premature differentiation and apoptosis, resulting in microcephaly (Wells et al. 2016). The organoids model of ZIKV infection also promotes the development of treatment. In a high-throughput drug screen of 6000 compounds, caspase-3 activity inhibitors, Emricasan and Niclosamide, were found to be effective in limiting ZIKV induced death of neural cortical progenitor and ZIKV replication (Xu et al. 2016). Except for ZIKV, *Japanese encephalitis virus* (JEV) infection, leading to Japanese encephalitis (JE), was modeled in generated telencephalon organoid (Zhang et al. 2018). Researchers found that JEV infection caused decline of cell proliferation and increase of cell death, and infected astrocytes and neural progenitors. In addition, they revealed variable antiviral immunity in brain organoids of different stages of culture, which also provide clues to develop effective therapeutics of such diseases.

Another example of PSCs-derived organoids application in human disease is with *hepatitis B virus* (HBV). In recent decades, treatments against HBV infection have improved; however, the development of personalized treatments has been hindered by the absence of personalized infection models. Nie and colleagues (Nie et al. 2018) generated PSCs-derived liver organoids that recapitulated the genetic background of the donor, and found HBV infection in PSCs-derived liver organoids could reproduce the life cycle of HBV and HBV-induced hepatic dysfunction, indicating that PSCs-derived liver organoids may provide a promising personalized infection model for the development of personalized treatment for hepatitis.

In recent years, ASCs-derived organoids have become the main force in infectious diseases modeling. ASCs-derived organoids can be adopted to model the viral infection of intestinal organoids. Gastric diarrhea in humans is mostly caused by *Human Norovirus (HuNoV)* and *Rotavirus* infection (Zheng et al. 2006). Although both of these viruses are rampant, no proper vaccine has been developed owing to the lack of *in vitro* culture method supporting their replication. Intestinal organoids that were cultured as monolayers allowed for extensive replication of multiple strains of noroviruses. For some strains, the addition of bile to the culture medium was required for replication (Ramani and Atmar 2014), indicating that not only are in vivo-like host cells required for productive infection but also an in vivo-like environment is relevant as well. Ettayebi and colleagues (Ettayebi et al. 2016) reproduced *HuNoV* infection in an organoid-virus co-culture system, with only a specific *GII.3 HuNov* strain requiring the presence of bile. Furthermore, lack of histo-blood group antigen (HBGA) expression in intestinal organoids limits *HuNov* replication, suggesting that this culture system allows the evaluation of potential treatments and preventions. Similarly, researchers have shown that *Rotavirus strain (simian SA11)* from clinical samples can proliferate in PSCs-derived intestinal organoids (Finkbeiner et al. 2012;Yin et al. 2015).

In urinary system, *BK virus*, which is a tubule-specific circular DNA virus, infects 1-10% of transplanted kidneys, leading in 10-80% of these infected kidneys to the loss of the donor organ and no curative treatment exists (Hirsch et al. 2005). Infection of kidney tubuloids (kidney-derived organoids in which only the tubular epithelium of the kidney is represented and glomeruli are lacking) with *BK virus* yielded a patchy infection with enlarged nuclei (due to intranuclear basophilic viral inclusions), similar to what is observed in kidney biopsies from patients with *BK virus* nephropathy (Bohl and Brennan 2007;Schutgens et al. 2019).

Respiratory infections pose a major global disease burdens (Ferkol 2014). *Respiratory syncytial virus* (RSV) alone causes hundreds of thousands deaths annually among children, mostly in developing countries (Nair et al. 2010). iPSCs-derived organoids of human airway epithelium can be infected by RSV virus, which can reproduce the morphological features of RSV infection in the distal lung (Chen et al. 2017). Persson and colleagues (Persson et al. 2014) established a ALI culture system for infection of human airway epithelium with RSV virus and they found that RSV has the potential to influence the cellular composition of the airway epithelium. Besides, ASCs-derived airway organoids using WENR methods can also be infected with RSV, recapitulating syncytia formation, cytoskeletal changes, and shedding of epithelial cells (Mueller et al. 2005). RSV-infected organoids attracted neutrophils more than did mock-infected control organoids, making this the first organoid model suitable for studying neutrophil-epithelium interactions (Sachs et al. 2019). Intriguingly, RSV infection strongly increased organoid motility and ultimately resulted in organoid fusion. Influenza viruses also pose a major public health problem worldwide, and novel emerging viruses may be lethal, as evidenced by the poultry-derived H7N9 virus infection that has had a 39% mortality rate since 2013. The infection of differentiated airway organoids with distinct strains of *influenza virus* can discriminate between poorly infective and highly infective strains (Zhou et al. 2018). Importantly, Hui and colleagues (Hui et al. 2018) compared human and avian strains of *influenza A virus* in *in vitro* human bronchus and airway organoids, and found that the infection of airway organoids yielded similar results regarding virus replication and cytokine response. In addition, infection of airway organoids with *enterovirus 71* (EV71) showed that EV71 replication kinetics are strain-dependent and the model help identify new infectivity makers for EV71 (van der Sanden et al. 2018).

The year of 2020 witnessed the outbreak of coronavirus disease-19 (COVID-19) caused by the virus severe acute respiratory syndrome-coronavirus 2 (SARS-CoV-2) which presents influenza-like symptoms ranging from mild disease to severe lung injury and multi-organ failure, eventually leading to death, especially in older patients with other co-morbidities. The WHO has declared that COVID-19 is a public health emergency of pandemic proportions. Organoids have been used as a great platform to research how COVID-19 affects human and causes damage and for identifying possible drug targets for COVID-19. Lamers and colleagues (Lamers and Beumer 2020) infected enterocytes in human small intestinal organoids with SARS-CoV and SARS-CoV-2 and found that the intestinal epithelium supports SARS-CoV-2 replication, and organoids can be served as an experimental model for coronavirus infection and biology. Zhou and colleagues (Zhou et al. 2020) established the first expandable organoid culture system of bat intestinal epithelium which were fully susceptible to SARS-CoV-2 infection and sustained robust viral replication. They also found active replication of SARS-CoV-2 in human intestinal organoids indicating that the human intestinal tract might be a transmission route of SARS-CoV-2. In addition, Monteil and colleagues (Monteil et al. 2020) found that SARS-CoV-2 can directly infect engineered human blood vessel organoids and human kidney organoids, which can be inhibited by human recombinant soluble ACE2 (hrsACE2), demonstrating that hrsACE2 can be a possible drug for early stages of COVID-19.

#### Bacterial disease

*Salmonella typhi (S. typhi)* and *Clostridium difficile (C. difficile)* are the two major bacterial intestinal pathogens that can cause diarrhea and gastrointestinal failures in humans. These pathogens infection has been successfully modeled using PSCs-derived intestinal organoids. Forbester and colleagues (Forbester et al. 2015) microinjected live *S. typhi* into the lumen of the iPSCs-derived intestinal organoids and revealed that upon injection, NF-κB signaling was activated and inflammatory factors were secreted, which was consistent with previous findings in animal models. Likewise, the Spence lab (Leslie et al. 2015) microinjected C. difficile toxin A (TcdA) and Toxin B (TcdB) into the lumen of iPSCs-derived intestinal organoids to model anaerobe *C. difficile* infection (CDI). The injection of TcdA recapitulates the impair of the epithelial barrier function and structure observed in organoids colonized with viable *C. difficile*. In another study, the Worrell lab (Engevik et al. 2015) observed a decreased expression of NHE3 (Sodium/Hydrogen Exchanger 3) and MUC2 (Mucoprotein2) protein in *C. difficile* infected organoids compared to normal organoids, which may help creating a favorable environment for its colonization.

Bacterium *Helicobacter pylori (H. pylori)* infection is a major risk factor for peptic ulcers, gastric adenocarcinoma and gastritis (Salama and Hartung 2013). Both gastric organoids derived from iPSCs and ASCs can be used to model *H. pylori* infection, by microinjecting *H. pylori* strain into the lumen of organoids (McCracken et al. 2014), which can ensure the apical side exposed to H. pylori. Luminal injection of H. pylori induces a potent NF-κB-mediated inflammatory response (Bartfeld et al. 2015), connecting excessive microbial colonization of *H. pylori* with the occurrence of gastric cancer. In a follow-up study, researchers adopted gastric organoids to find out how *H. pylori* finds its gastric niche: a potent chemoattractant, urea, which produced by gastric epithelium is essential for the colonization of *H. pylori* in the gastric mucosa (Huang et al. 2015a).

Chronic *Salmonella* infection of the gall bladder is associated with gallbladder carcinoma (Shukla et al. 2000). Scanu and colleagues (Scanu et al. 2015) showed that after *Salmonella* infection, mouse gallbladder organoids exhibited characteristics of loss of polarity, familiar with those showed in the mouse model of gallbladder cancer. Another study found that gallbladder organoids pre-exposed to *Salmonella* that lack functional TP53 showed neoplastic transformations by activating AKT (protein kinase B) and MAPK (mitogen-activated protein kinase) pathway and could grow in culture media free of growth factors (Scanu et al. 2015).

#### Parasitic disease

The protozoan parasite *Cryptosporidium* causes life-threatening diarrhea in immunocompromised individuals (e.g. people living with HIV and malnourished children), and infection may spread to the lungs (Checkley et al. 2015). Drug development requires detailed pathophysiology information of *Cryptosporidium*, but the lack of an optimal *in vitro* culture system hinders the experimental approaches. Heo and colleague (Heo et al. 2018) infected epithelial organoids derived from human small intestine and lung with *Cryptosporidium* and found that the parasite can reproduce within the organoids and complete its complex asexual and sexual life cycles for multiple rounds.

*Plasmodium* parasites can cause malaria, which poses a significant global health burden, with over 200 million cases every year. *Plasmodium* parasites are maintained between Anopheles mosquitoes and mammalian hosts in a complex life cycle, and models to study them are challenging to establish, particularly for *Plasmodium* species that infect humans (Mellin and Boddey 2020). Recently, several studies reported the application of iPSCs-derived hepatocyte-like cells to model in vitro liver stage infections with P. berghei, P. yoelii, P. falciparum, and P. vivax (Ng et al. 2015). It was found that P. yoelii and P. falciparum infections of organoids recapitulated the primaquine sensitivity found in vivo. Chua and colleagues (Chua et al. 2019) infected organoids derived from simian and human hepatocytes, with *P. cynomolgi* and *P. vivax* and found that organoids could support the complete liver stage of both simian and human parasites, from initial infection with sporozoites, to the release of merozoites capable of erythrocyte infection. This study also illustrated the use of infected organoids to evaluate the response to an anti-relapse drug, highlighting the potential for organoids as a parasite drug screening platform, particularly in parasites with life-cycles longer than their host cells.

### Cancer

#### Colorectal cancer

Though organoids derived from tumor and matched normal epithelial tissues provide valuable research tools for cancer biology, one of the most remarkable improvements in organoid research is the capacity to manipulate the genomes, transcriptomes and epigenomes of normal epithelial organoids to study the role of specific alterations in the process of tumorigenesis.

Murine organoid cultures were firstly used to study the early stages of tumorigenesis. Li and colleagues (Li et al. 2014) adopted the ALI culture approach combined with genetically engineered mouse model and the retrovirus-mediated delivery of shRNA constructs, to model multi-step tumorigenesis in organoids derived from digestive tract, including the colon, stomach, and pancreas. Pancreatic and gastric organoids exhibited dysplasia as a result of expression of *Kras*^*G12D*^, *p53* loss or both. While colon organoids needed assembled *Apc*, *p53*, *Kras*^*G12D*^ and *Smad4* mutations for malignant transformation to invasive adenocarcinoma-like morphology. All engineered organoids presented histologic characteristics of adenocarcinoma after subcutaneous implantation was performed to immunocompromised mice.

**Fig. 9:**
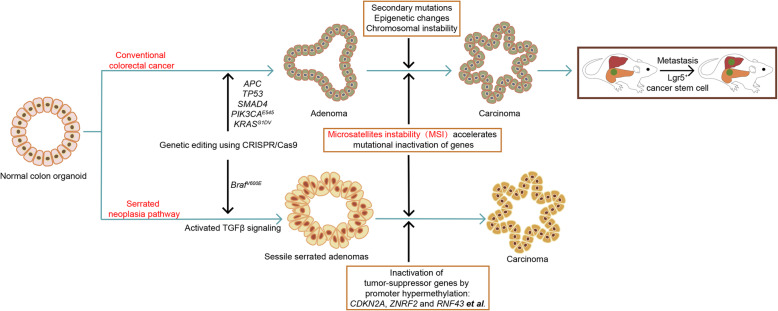
Assessing different tumorigenicity and metastases mechanisms of combinative mutations in colon cancer based on normal colon organoids by using CRISPR-Cas9 technology

Following research tried to model multi-step tumorigenesis of conventional CRC, which is characterized by chromosomal instability (CIN) (Fig. 9). Drost and colleagues (Drost et al. 2015) adopted CRISPR-mediated knock-out of the tumor suppressors *APC*, *TP53*, and *SMAD4*, married with CRISPR-mediated knock-in of the oncogenes *KRAS*^*G12D*^ to model multi-step tumorigenesis. After being selected by niche factors in the culture media, cultures of organs were successfully built with complex oncogenic multi-gene modules that contain up to four simultaneous changes. The 4-hit AKST (*APC, KRAS*^*G12D*^*, SMAD4, and TP53*) organoids could grow without stem cell niche factors such as Wnt-3, R-spondin-1 and EGF. AKST organoids were able to generate tumors with characteristics of invasive carcinoma upon subcutaneous implantation into immunocompromised mice. Matano and colleagues (Matano et al. 2015) applied a similar method to model tumorigenesis by inserting an additional CRISPR-mediated knock-in of the oncogene *PIK3CA*^*E545*^*,* in addition to AKST. Both studies showed that organoids with *APC* and *TP53* mutations showed extensive aneuploidy, which is the hallmark of the CIN pathway.

Xenotransplantation of engineered colorectal tumor organoids makes it possible to study cancer stem cells *in vivo* (de Sousa e Melo et al. 2017;Shimokawa et al. 2017) and leads to metastatic diseases, making organoids a useful research tool to study metastasis mechanisms (Fumagalli et al. 2017;Roper et al. 2017). de Sousa e Melo and colleagues (de Sousa e Melo et al. 2017) combined CRC mouse with the *Lgr5*^*DTR/eGFP*^ allele. The resulting animals carry two of the most frequently mutated genes, *Apc* and *Kras*^*G12D*^, and in addition, express a diphtheria toxin receptor fused to an eGFP under the endogenous regulatory region of Lgr5, allowing specific elimination and visualization of Lgr5-positive stem cells. Using this model, it was found that in the absence of cancer stem cells, liver metastases did not occur, whereas primary tumors did not regress, indicating that Lgr5-positive cancer stem cells are required for metastasis. In another study, Fumagalli and colleagues (Fumagalli et al. 2017) orthotopically transplanted CRISPR-mediated *KRAS, APC, TP53*, and *SMAD4* co-mutated human colon organoids into mice and showed that metastases to the liver and lungs occurred in 44% of the mice. Almost no metastasis occurred when organoids carrying mutations in only three of these four genes were transplanted to mice; however, the lack of the fourth mutation could be overcome by providing the niche factor upstream of the absent mutation. For example, organoids with triple mutants lacking *SMAD4* inactivation metastasized when Noggin was added to the cells. These findings indicate that metastatic potential is directly related to the loss of niche factor dependency.

CRC that arises from the serrated neoplasia pathway is different from CRC that arises from the conventional CIN pathway. The activation of oncogene *BRAF* initiated the serrated pathway, followed by the extensive hypermethylation of CpG island methylator phenotype, and subsequent inactivation of tumor suppressor genes (Bae and Kim 2016). Fessler and colleagues (Fessler et al. 2016) firstly built the organoids to model the serrated pathway of CRC by introducing the *BRAF*^*V600E*^ mutation into normal human colon epithelial organoids via homologous recombination. They revealed that induction of a mesenchymal phenotype upon TGFβ treatment prevails in the *BRAF*^*V600E*^ mutated organoids generating sessile serrated adenomas (SSAs). In a recent study, by analyzing the genomic data from TCGA, CRC-associated gene alterations including *Braf*^*V600E*^, *Cdkn2a*, *Tgfbr2*, *Znrf2* and *Rnf43,* were selected to be introduced into murine colon organoids using CRISPR-Cas9 technology to model the serrated pathway (Lannagan et al. 2019). Upon subcutaneous implantation into immunocompromised mice, these engineered organoids could generate tumors with characteristics of serrated CRC, including desmoplastic stromal responses, infiltrative growth, mucinous differentiation, tumor budding, and the formation of colon tumors spontaneously metastasizing to the liver. To be pointed out, transplantation of *Braf*-mutated organoids failed to generate tumors, while injection of organoids with both mutations of *Braf* and *Tgfbr2* led to invasive adenocarcinoma. Implantation of organoids with those and additional mutations (*Cdkn2a*, *Znrf2* and *Rnf43*) resulted in increased tumor initiation and decreased survival time. Moreover, to study the development of the R-spondin-driven serrated pathway, another study also introduced CRISPR-mediated gene fusions on *BRAF*^*V600E*^ and/or *TP53* accompanied with the R-spondin fusion EIF3E (Eukaryotic Translation Initiation Factor 3 Subunit E )-RSPO2 to normal human colon epithelial organoids (Kawasaki et al. 2020). The resulting *BRAF*^*V600E*^ organoids showed aberrant crypt formation ability with poor engraftment capacity, while organoids with mutations of *BRAF*^*V600E*^ and *TP53* with EIF3E-RSPO2 fusion generated flatly elevated lesions and hyperplastic crypt structures with ‘V’-shaped serration and basal dilation, together with enhanced engraftment capacity compared with only *BRAF*^*V600E*^ mutated organoids.

The loss of DNA mismatch repair enzymes, such as MutL homolog 1 (MLH1), is common in CRCs, resulting in tumors with high mutational load. These tumors are characterized by microsatellite instability (MSI), as repetitive short sequences in the genome, which leads to changes in copy number following the loss of mismatch repair enzymes. Drost and colleagues (Drost et al. 2017) used CRISPR-mediated knock-out of MLH1 and cultured organoids for 2 months to allow accumulation of mutations. Subsequent DNA analyses of cultured organoids revealed an increase in mutational load compared with controls, which was similar to that of MSI colorectal tumors. This study shows that organoids faithfully recapitulate *in vivo* mutagenesis and allow for the identification of mechanisms of tumor development.

In addition, rare type of colorectal cancer can also be modeled based on organoid culture. Li and colleagues (Li et al. 2019) have established a novel organoid line of colon signet-ring cell carcinoma (SRCC), which accounts for only 1% of colorectal cancers. The novel organoid line resembles the primary tumor histologically and molecularly, and can efficiently generate tumors on xenografts. Targeted DNA sequencing with drug screening of 88 compound identified that JAK2 (Janus Kinase 2) might be a potential treatment target. An *in vitro* drug screening experiment exhibited that SRCC organoids can be sensitively treated by AT9283 and Pacritinib, two JAK2 inhibitors, which was consistent with the *in vivo* xenograft response. The study provides a novel *in vitro* research tool for colorectal SRCC and sets an example for personalized medicine based on organoids for rare cancer.

#### Pancreatic cancer

Pancreatic ductal adenocarcinoma (PDAC) accounts for about 90% of all pancreatic malignancies and over 90% PDAC patients harbor activation of the oncogene *KRAS*. *KRAS* can consequently induce inactivation of various tumor suppressor genes including *TP53*, *CDKN2A*, *SMAD4* and *BRCA2* to accelerate PDAC development and progression (Kanda et al. 2012;Morris and Wang 2010). Establishment of murine pancreatic organoids bearing a lox-stop-lox (LSL) *KrasG12D* allele provided valuable insights for the development of PDAC. In one study (Li et al. 2014), pancreatic organoids derived from *LSL-Kras*^*G12D*^ (K), *Tp53*^*flox/flox*^ (P), and *LSL-KRas*^*G12D*^; *Tp53*^*flox/flox*^ (KP) mice were successfully built using ALI method. K, P, and KP organoids exhibited *in vitro* dysplasia and increased invasive behavior, and could *in vivo* generate well differentiated to poorly differentiated adenocarcinoma upon implantation into immunocompromised NOG mice. KP organoids had most poorly differentiated morphology, with significant loss of E-cadherin, indicating increased epithelial to mesenchymal transition. Researchers of another study generated low-grade preinvasive pancreatic intraepithelial neoplasias (PanINs) *in vivo* by crossing the murine *LSL-Kras*^*G12D*^ allele to a pancreas-specific *Pdx1-Cre* driver and organoids derived from PanIN lesions could be long-term expanded a produce PanIN-like lesions that persisted for up to 2 months upon transplantation (Boj et al. 2015).

Two studies have adopted CRISPR-Cas9 technology to manipulate PDAC driver genes including *KRAS*^*G12V*^, *CDKN2A*, *TP53* and *SMAD4* (KCTS) in normal human pancreatic ductal organoids (Lee et al. 2017;Seino et al. 2018). Upon orthotopically transplantation to immunocompromising mice, different combinations of mutants in organoids exhibited distinct PanINs features, but only KCTS organoids displayed PDAC histopathological transformation (Seino et al. 2018). Notably, KC and KT organoids died after WNT removal for 1-3 weeks, while KCT and KCTS organoids survived and expanded for at least 3 months, suggesting that *CDKN2A* and *TP53* mutations are essential for organoids to grow independently of stem cell niche factors (Seino et al. 2018). In all, these studies indicate that organoids combined with CRISPR-mediated sequential mutations can recapitulate tumorigenesis and progression from PanIN to PDAC. Moreover, organoids could also be used to explore the roles of various factors such as the redox regulator NRF2 and the transcriptional enhancer FOXA1 in PDAC progression (Chio et al. 2016;Roe et al. 2017). Further researches are need to apply the improved knowledge of molecular mechanisms to clinics.

#### Gastric cancer

Gastric cancer are classified into four molecular subtypes based on deep sequencing: Epstein-Barr virus positive, MSI, CIN and genomically stable (Cancer Genome Atlas Research 2014). Nanki and colleagues (Nanki et al. 2018) adopted organoids to illustrate the genotype-phenotype associations of gastric cancer. Phenotype analyses of organoids derived from gastric cancer patients indicated multiple genetic and epigenetic ways to confer Wnt and R-spondin niche independency. They found that induction of *RNF43* and *ZNRF43* mutations was sufficient for gastric cancer organoids to gain R-spondin independency. The phenomenon was then validated in CRISPR-Cas9 engineered gastric organoids. In a similar study, Seidlitz and colleagues (Seidlitz et al. 2019) generated human and murine gastric cancer organoids which recapitulated the typical features and altered pathways of each of four molecular subtypes of gastric cancer. The combination of organoids and CRISPR-Cas9 technologies promotes research on the molecular mechanisms of gastric cancer tumorigenesis and progression, thereby accelerating the development of preclinical gastric cancer models for novel drug development and personalized medicine.

#### Breast cancer

Recently, Dekkers and colleagues (Dekkers et al. 2019) attempted to model multi-step carcinogenesis in breast epithelial organoids derived from human reduction mammoplasties using CRISPR-Cas9 technology. They introduced CRISPR-mediated knock-out of four breast cancer-associated tumor suppressor genes, including *P53, PTEN, RB1 and NF1* (PTRN) to mammary progenitor cells. Mutated organoids could long-term expanded and generated ER+ luminal tumors upon transplantation into mice for 1 out of 6 PTR-mutated and 3 out of 6 PTRN-mutated organoid lines. These organoids had various response to endocrine therapy or chemotherapy, indicating the potential application of this model to facilitate our understanding of the molecular mechanisms in specific subtypes of breast cancer.

#### Prostate cancer

In prostate cancers, 40-80% of tumors harbor a gene fusion between the androgen receptor (AR)-responsive transmembrane protease serine 2 (*TMPRSS2*) gene and E26 transformation-specific (ETS) family transcription factor, most often the oncogene ETS-related gene (ERG) (Tomlins et al. 2005). TMPRSS2 and ERG, both located on chromosome 21, are separated by about 3 million base pairs. Using CRISPR-Cas9, a TMPRSS2-ERG fusion was successfully introduced into mouse prostate organoids using a template that brought these two DNA regions together. This genetic alteration resulted in AR-mediated overexpression of ERG, an effect that can be prevented by androgen receptor antagonist, consistent with those seen *in vivo* (Driehuis 2017).

## Organoids for translational research

### Living organoid biobanks as a tool for personalized treatment and drug development

As mentioned above, organoids can be efficiently established from patient-derived normal and tumor tissue samples, which can be cryopreserved and stored in living organoid biobanks. PDOs resemble the tumor epithelium they were derived from both phenotypically and genetically. However, molecular profile alone is not sufficient to adequately predict drug sensitivity. Even in patients with the same genotype, drug response varies. Besides, some mutations are rare, thus clinical trials are impossible and efficacy testing is required for drugs to be conducted on an individual basis. Thus, combined molecular and therapeutic profiling of PDOs may help predict treatment response and contribute to personalized cancer treatment and drug development.

#### Gastrointestinal cancer

A number of organoid biobanks have been reported since 2014 (Table 3). A colon cancer derived biobank of 22 lines was established in 2015, setting an example for a standard biobank (van de Wetering et al. 2015). All samples performed RNA sequencing and whole genome sequencing analysis. The molecular characteristics of PDOs cover all five consensus molecular subtypes of CRC. The mutations in the organoids were largely concordant with the original tumors, which was validated in a set of organoids established of colorectal metastases (Weeber et al. 2015). High-throughput screening of a panel of 83 compounds found that differences in drug sensitivity among the organoid lines that in some cases correlated with specific mutation. For example, RNF43-mutant organoids were sensitive to WNT secretion inhibitors, and KRAS-mutant organoids were resistant to the EGFR (epidermal growth factor receptor) inhibitors, including cetuximab and afatinib.

**Table 3:** List of biobanks of different tumor types

Organ of origin	Number of lines ^a^	Histological subtypes	Efficiency	Year	Ref.
Colon	22	Adenocarcinomas	90%	2015	(van de Wetering et al. 2015)
Colorectum	8	Colorectal metastases	71%	2015	(Weeber et al. 2015)
Colorectum	55	Premalignant lesions (tubular and tubulovillous adenomas, sessile serrated lesions, and a hyperplastic polyp) Adenocarcinomas (well differentiated, moderately differentiated, poorly differentiated, mucinous, not specified) Metastases of adenocarcinomas Neuroendocrine carcinomas	100%	2016	(Fujii et al. 2016)
Colorectum	35	NR	60%	2017	(Schütte et al. 2017)
Colorectum	34	Colorectal metastases	63%	2019	(Ooft et al. 2019)
Rectum	71	Cystic fibrosis	NR	2016	(Dekkers et al. 2016)
Rectum	65	Adenocarcinoma	77%	2019	(Ganesh et al. 2019)
Rectum	80	locally advanced rectal adenocarcinoma	85.7%	2019	(Yao et al. 2020)
Pancreas	8	Ductal adenocarcinomas	80%	2015	(Boj et al. 2015)
Pancreas	17	Ductal adenocarcinomas	85%	2015	(Huang et al. 2015b)
Pancreas	39	Ductal adenocarcinomas	88%	2018	(Seino et al. 2018)
Pancreas	114	Ductal adenocarcinomas	75%	2018	(Tiriac et al. 2018)
Pancreas	30	Pancreatic ductal adenocarcinoma Acinar cell carcinoma Cholangiocarcinoma Adenosquamous Pancreatic ductal adenocarcinoma Intraductal papillary mucinous neoplasm-derived Pancreatic ductal adenocarcinoma Papilla of Vater adenocarcinoma	60%	2019	(Driehuis et al. 2019c)
Liver	8	Hepatocellular carcinoma Cholangiocellular carcinoma	100%	2017	(Broutier et al. 2017)
Liver	13	Hepatocellular carcinomaCholangiocellular carcinomaLymphoepithelioma-like cholangiocarcinoma	33%	2017	(Nuciforo et al. 2018)
Biliary Tract	6	Intrahepatic cholangiocarcinomaPancreatic ductal adenocarcinomaGallbladder cancerNeuroendocrine carcinoma	42%	2019	(Saito et al. 2019)
Prostate	7	Adenocarcinoma metastases Circulating tumor cells	15-20%	2014	(Gao et al. 2014)
Bladder	22	Papillary urothelial carcinomaUrothelial carcinomaSquamous cell carcinoma	70%	2018	(Lee et al. 2018)
Breast	95	Ductal carcinoma Lobular carcinoma	80%	2018	(Sachs et al. 2018)
Ovary	33	High-grade serous carcinoma	100%	2018	(Hill et al. 2018)
Ovary	56	Borderline tumors (both mucinous and serous) Clear cell carcinomas Endometrioid carcinomas Mucinous carcinomas Low-grade serous carcinomas High-grade serous carcinomas	65%	2019	(Kopper et al. 2019)
Head and Neck	31	Squamous cell carcinoma	60	2019	(Driehuis et al. 2019a)
Brain	70	Glioblastoma	91.4%	2020	(Jacob et al. 2020)
Mixed^b^	56	Tumors from prostate, breast, colorectal, esophagus, brain, pancreas, lung, small intestine, ovary, uterus, soft tissue (not further specified), bladder, ureter, kidney	38.6%	2017	(Pauli et al. 2017a, 2017b)
Mixed	62	Metastatic colorectal cancer Metastatic gastroesophageal cancer Metastatic cholangiocarcinoma	70%	2018	(Vlachogiannis et al. 2018)
Mixed (ALI)	49	Tumors from lung (adenocarcinoma and squamous cell carcinoma), kidney (clear cell carcinoma, papillary carcinoma, Wilms tumor and chromophobe carcinoma) and thyroid (papillary carcinoma)	76%	2018	(Neal et al. 2018)

*NR* Not reported

^a^Refers to the number of organoid lines reported not the number of patients (for some patients, multiple lines were established)

^b^Histological types were not comprehensively reported

Later, Schutte and colleagues (Schütte et al. 2017) reported a biobank of 35 organoid lines from CRC. They found that organoid models reproduce most the genetic and transcriptomic characteristics of the donors, but determined less complex molecular subtypes for the absence of stroma. Drug screening with therapeutic compounds representing the standard of care for CRC, combined with molecular profiles, helped identify a signature outperforming RAS/RAF mutation which has predictive value for sensitivity to the EGFR inhibitor cetuximab.

Drug response in organoids and clinical response was also observed to prove that the *in vitro* organoid response correlates with the *in vivo* response. A clinical study of PDOs derived from metastatic gastroesophageal and CRC showed a strong correlation (100% sensitivity, 93% specificity, 88% positive predictive value, and 100% negative predictive value) between the *in vitro* organoid response to a set of targeted therapies and chemotherapies and the response of the tumor in patients (Vlachogiannis et al. 2018). Another study adopted organoids for colon cancer chemoprediction showing that PDOs test predicted more than 80% of patients’ response treated with irinotecan-based therapies (Ooft et al. 2019). Together, these studies indicate the potential of tumor-derived organoids to predict patients’ responses.

Recently, two studies showed the applications of PDOs derived from rectal cancer to predicting patient responses to neoadjuvant chemoradiation therapy. Yao and colleagues (Yao et al. 2020) generated a rectal cancer derived biobank (N=80) and tested PDOs’ sensitivity to 5-FU, irinotecan, or radiation. They incorporated a correlation between *in vitro* responses in organoids and the histopathologically determined tumor regression scores (TRGs) after surgical resection to define prognostic cut-offs. Using these parameters, the *in vitro* responses could predict clinical responses with an impressive area under the curve (AUC) of 0.88 and an accuracy of 84%. In the other study, Ganesh and colleagues (Ganesh et al. 2019) established 65 PDO lines from rectal cancer to test responses to neoadjuvant chemoradiation therapy, including the standard FOLFOX chemotherapy and radiation. The PDO responses significantly reflected the patients’ progression-free survival. Moreover, PDOs generated invasive rectal cancer followed by metastasis upon transplantation into murine rectal mucosa, exhibiting the same *in vivo* metastatic route as in the corresponding patients.

For pancreatic cancer, Boj and colleagues (Boj et al. 2015) were the first to successfully developed organoids from patient-derived PDACs. Subsequently, Seino and colleagues (Seino et al. 2018) generate an extensive organoids biobank of PDACs (N=39) covering both classical and basal subtypes according to gene expression signatures. They found basal-type PDACs derived organoids are more independent of Wnt signaling, which are more invasive and aggressive clinically, indicating that progression of PDACs are accompanied by loss of stem cell niche dependence. Recently, Tiriac and colleagues (Tiriac et al. 2018) generated a much larger PDAC biobank (N=114) and exposed a subset of these organoid lines to the standard-of-care chemotherapies. Their sensitivities paralleled clinical responses in patients. Besides, gene expression signatures of chemosensitivity based on organoids were developed to help predict responses to chemotherapy in both the adjuvant and advanced disease settings. By high throughput drug screening, they nominated alternative treatment strategies for chemo-refractory PDO. Another study also used PDOs (N=30) to identify novel therapeutics to target pancreatic tumor cells in a biobank covering different histological subtypes, including PDACs, acinar cell carcinoma, cholangiocarcinoma, adenosquamous-PDACs, intraductal papillary mucinous neoplasm (IPMN)-derived PDACs and papilla of Vater adenocarcinomas (Driehuis et al. 2019c). PDOs were exposed to 76 therapeutic agents currently not exploited in the clinic. The PRMT5 (Protein Arginine Methyltransferase 5) inhibitor, EZP015556, was shown to target MTAP (methylthioadenosine phosphorylase)-negative tumors, but also appeared to constitute an effective therapy for a subset of MTAP-positive tumors, indicating the importance of personalized approaches for cancer treatment.

Huch and colleagues (Broutier et al. 2017) described a liver tumor biobank (N=13) containing hepatocellular carcinoma and cholangiocarcinoma, as well as the rarer lymphoepithelioma-like cholangiocarcinoma. In drug screening experiments with 29 compounds, the ERK (extracellular regulated protein kinases) inhibitor SCH772984 was found to effectively inhibit the growth of tumor organoids, which was validated *in vivo* using xenotransplanted organoid lines in mice, highlighting SCH772984 as a possible therapeutic agent.

Biliary tract carcinomas-derived organoids biobank was also established, covering intrahepatic cholangiocarcinoma, gallbladder cancer, and neuroendocrine carcinoma of the ampulla of Vater (Saito et al. 2019). Gene expression profiling of the organoids indicated that *SOX2, KLK6* and *CPB2* could be potential prognostic biomarkers. Drug screening using a compound library of 339 drugs showed that the antifungal drugs, amorolfine and fenticonazole, significantly suppressed the growth of biliary tract carcinomas organoids with little toxicity to normal biliary epithelial cells.

#### Genitourinary cancer

The organoids biobank of metastatic prostate cancer covering both AR (androgen receptor)-positive and -negative subtypes was the first reported biobank established by Gao and colleagues (Gao et al. 2014). The biobank captured the most common genetic aberrations in prostate cancer, including *TMPRSS2–ERG* fusion, homozygous deletions of *PTEN* and *CHD1*, as well as typical copy number variations. To be pointed out, organoids derived from circulating tumor cells were also successfully established in this biobank, showing that at least in some cases, organoids can be established from less invasive blood samples.

A bladder cancer derived organoids biobank (N=20) was established containing urothelial carcinomas and one squamous cell carcinoma (Lee et al. 2018). Organoid lines were interconvertible with orthotopic xenografts and recapitulated the mutational spectrum of the corresponding tumor type, including activation of *FGFR3* and mutations in epigenetic regulators such as ARID1A. Drug screening of 40 compounds based bladder tumor organoids showed partial correlations with mutational profiles as well as treatment resistance, and the drug responses can be validated using xenografts *in vivo*.

#### Women’s cancers

A biobank of breast cancer organoids (N=95) has been described covering major histological subtypes (invasive ductal carcinoma and invasive lobular carcinoma) and all molecular subtypes based on gene expression (Sachs et al. 2018). Organoid morphologies matched the histopathology of the original tumors, and hormone receptor [estrogen receptor (ER), progesterone receptor (PR)], HER2 status and copy number variations were retained. ER and PR status have predictive value for the outcome of endocrine therapy (e.g. tamoxifen), while HER2 is a target for targeted therapy (e.g. trastuzumab) and also has predictive value for chemotherapy outcome. The response of breast cancer-derived organoids to HER2 inhibitor afatinib and to endocrine therapy tamoxifen were consistent with *in vivo* xeno-transplantations and patient response in clinic.

An organoid biobank of high-grade serous ovarian cancer (HGSC) (N=33) was established by Hill and colleagues (Hill et al. 2018). Up to 50% of all patients with HGSC have DNA repair defects, typically mutation of *BRCA1* or *BRCA2*. These patients were thought to benefit from treatment with poly (ADP-ribose) polymerase (PARP) inhibitors. In the clinical setting, mutation analysis alone is not sufficient to adequately predict drug sensitivity. The study showed that functional assays in organoids are a better predictor than the genomic analysis in clinic, implying that functional assays in organoids may improve the prediction of drug sensitivity beyond what can be achieved with genomic analysis alone. Kopper and colleagues (Kopper et al. 2019) established a second ovarian cancer biobank (N=56) that captured all of the main histological subtypes, including borderline tumors, endometroid carcinomas, mucinous carcinomas, LGSC and HGSC. Notably, a novel single-cell DNA sequencing method was used to demonstrate intra-patient heterogeneity was preserved in organoids when compared with the original tumor. PDOs can be used for drug-screening analyses and capture distinct responses of different histological subtypes to platinum-based chemotherapy, including acquisition of chemoresistance in recurrent disease. Besides, PDOs can also be xenografted, enabling *in vivo* drug sensitivity analyses. Taken together, PDOs of ovarian cancer have potential application for translational research and precision medicine.

#### Head and neck cancer

Driehuis and colleagues (Driehuis et al. 2019a) established an organoid biobank (N=31) derived from head and neck squamous cell carcinoma (HNSCC). PDOs recapitulates genetic and molecular characteristics of original HNSCCs and can generate tumors upon transplantation to immunocompromised mice. The authors observe different responses to commonly used drugs in clinic including cisplatin, carboplatin, cetuximab, and radiotherapy *in vitro*. Besides, drug screens exhibit selective sensitivity to targeted drugs that are not normally used in clinic for patients with HNSCC. These findings may inspire the personalized treatment of HNSCC and expand the list of HNSCC drugs. In another study, the authors reported PDOs derived from HNSCC can also be used to evaluate their response to targeted photodynamic therapy and to ensure the safety of the treatment at the same time by testing it on organoids derived from matched normal tissues (Driehuis et al. 2019b).

#### Glioblastoma

All organoids discussed above were derived from tumors of epithelial origin, known as carcinomas. Recent advances show organoids derived from primary glioblastoma tissue, setting the stage for growing organoids from non-epithelial tumors (Hubert et al. 2016). Glioblastoma presents great heterogeneity, and thus it’s difficult to generate an ideal *in vitro* model which can recapitulate the *in vivo* situation of the donor. By modifying the method to develop cerebral organoids, the PDOs can be successfully derived both from primary lesion of glioblastoma and from brain metastases. Once formed, PDOs presented hypoxia gradients and mimicked cancer stem cell heterogeneity with rapidly dividing outer cells surrounding the hypoxic core of differentiated cells and diffuse, quiescent cancer stem cells. Drug testing based on organoids showed that non-stem cells were sensitive to radiation therapy, whereas adjacent cancer stem cells were radioresistant. Orthotopic transplantation of PDOs resulted in tumors recapitulating histological features of the parental tumor.

Based on this method, Jacob et al. (2020) established a larger organoid biobank derived from glioblastoma (N=70), recapitulating the histological characteristics, cellular diversity, gene expression, and mutational profiles of their donors. The organoids generated rapid, aggressively infiltrated tumors upon transplantation into adult rodent brains. The authors observe different responses to exposure of radiation with concurrent temozolomide, which was consistent with the patients’ response and survival in clinic. Notably, the authors further demonstrate the utility of organoids in modeling immunotherapy by co-culture of chimeric antigen receptor T (CAR-T) cells with organoids. They observed specific tumor cells were targeted and killed by CAR-T cells. The study expands the application of PDOs in personalized treatment to include immunotherapy.

#### Mixed tumors

Several biobanks containing organoids derived from mixed cancers were established for pan-cancer research. Pauli et al. (2017a, 2017b) developed a robust precision cancer platform, by integrating whole exome sequencing with a living biobank which allows high throughput drug screens on PDOS. The biobank included tumors derived from prostate, breast, colorectum, esophagus, brain, pancreas, lung, small intestine, ovary, uterus, soft tissue, bladder, ureter and kidney. In another study, to model tumor immune microenvironment, Kuo and colleagues (Neal et al. 2018) established PDOs based on ALI method from >100 individual patient tumors of 19 distinct organs and 28 histological subtypes. These PDOs included common cancers such as colon, pancreas, and lung, and rarer histologies such as bile duct ampullary adenocarcinoma, brain schwannoma, and salivary gland pleomorphic adenoma. PDOs in this biobank retain immune cells and should enable immuno-oncology investigations and facilitate personalized immunotherapy testing.

#### Cystic fibrosis

As mentioned above, CF is a lethal genetic disease caused by CFTR mutations that impairs the function of many organs including the intestine, lung, pancreas, sweat gland, liver, and kidney. The disease is characterized by the buildup of viscous, sticky mucus which clogs airways, causes chronic digestive system problems and leads to CF-related diabetes. Approximately 2,000 CF-causing mutations of CFTR have been described, and drug efficacy varies among the different genotypes (Cutting 2015). Thus, there is a need for a personalized medicine approach to predict treatment response.

**Fig. 10:**
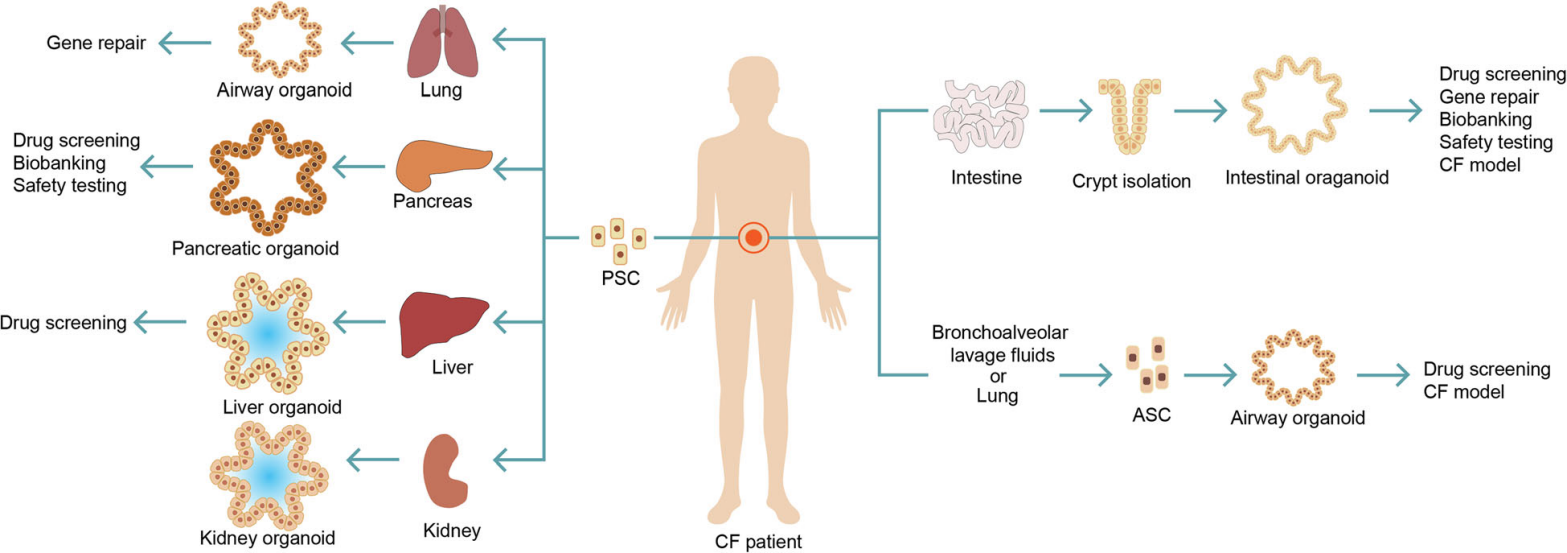
Organoids for modeling cystic fibrosis (CF) and its multiple applications. Organ-specific pathologies of CF can be studied separately using organoids derived from distinct tissues and applied to personalized drug screening

Beekman and colleagues (Dekkers et al. 2016) first established an organoid biobank derived from rectum of 71 CF patients with 28 different CFTR genotypes. Based on the biobanking, they developed a personalized medicine approach by using FSK-induced swelling assay to select clinical responders to CF modulators. Two patients with the rare and uncharacterized F508del/G1249R genotype responded *in vitro* to a specific CF modulator, ivacaftor (KALYDECO, Vertex Pharmaceuticals). The responses were consistent with their *in vivo* clinical response to the treatment, reflected by improved pulmonary function and sweat chloride tests. In a prospective follow-up study involving 24 participants (Berkers et al. 2019), the predictive power of the FSK assay was further substantiated, as the *in vitro* assay correlated with changes in pulmonary function and sweat chloride tests conducted *in vivo*. Besides CF-rectal organoids, pancreatic organoids and cholangiocyte-like organoids derived of CF-iPSCs have also shown the potential for drug screening (Sampaziotis et al. 2015;Simsek et al. 2016). In the Netherlands, the licensing of ORKAMBI (lumacaftor/ivacaftor, Vertex Pharmaceuticals) allows treatment of CF patients solely based on a positive organoid swelling response, demonstrating the potential of organoid-based assays for delivering personalized medicine (Fig. 10).

In summary, organoid biobanks have been established for multiple tumor types (Table 3), including non-epithelial glioblastoma, and several principals can be concluded. First, PDOs can be established from small biological samples, such as biopsies and even circulating tumor cells, and these organoids can generate tumors upon xenotransplantation. Second, the PDOs in biobanks recapitulate histological and genetic aspects of the original tumors, which holds not only for localized primary tumors but also for metastatic tumors. Third, high-throughput drug screening experiments in organoids correlate with the response in patients and lead the identification of new therapeutic targets. Combining genetic sequencing with functional assays in PDOs will facilitate personalized treatment, even including immunotherapy, which will be discussed in detail in Section 3.4.

### Genetic repair in organoids

Schwank and colleagues (Schwank et al. 2013) firstly demonstrated that it is feasible to repair genetic defects in organoids. Intestinal organoids derived from two CF patients with a homozygous CFTR F508 deletion were repaired using CRISPR-Cas9 technology. After repair, FSK-induced swelling was restored, functionally demonstrating CFTR activity. Later, Firth and colleagues (Firth et al. 2015) generated iPSCs from CF patients with a homozygous deletion of CFTR F508 and corrected this mutation using CRISPR technology to target corrective sequences to endogenous CFTR genomic locus, combining with selection system. The corrected iPSCs-organoids were able to differentiate mature airway epithelial cells with normal CFTR expression and function.

However, CFTR gene repair in organoids and subsequent transplantation into patients is hard to be applied in the clinic. First, the loss of CFTR functions results in disease in multiple organ systems, which would require the transplantation of organoids into multiple tissue sites. Second, a high percentage of repaired cells per organ would be required for functional restoration. Third, ethics problems in organoids transplantation are controversial and needs to reach a consensus in the research field.

### Organoids transplantation

Human and murine organoids have been orthotopically transplanted into mice to model disease or to show tumorigenic potential. Here, we discuss studies that used organoid transplantation as therapy.

Yui and colleagues (Yui et al. 2012) firstly exploited the feasibility to apply organoids to repair damaged epithelium. GFP^+^ murine colon organoids derived from Lgr5^+^ stem cells were reintroduced into mice with DSS (Dextran Sulfate Sodium Salt) -induced acute colitis. Transplanted cells readily integrated into the mouse colon and covered superficially damaged tissue. 4 weeks after transplantation, the donor cells constituted a single-layered epithelium, which formed functionally and histologically normal crypts that were able to self-renew. Further, engrafted mice had higher body weights than ungrafted ones, indicating that donor cells contributed to the recovery of DSS-induced acute colitis. Although further optimization is still needed, the current study indicates that *in vitro* expansion and transplantation of organoids may be a promising treatment choice for patients with severe gastrointestinal epithelial injuries.

Orthotopic transplantation of liver organoids has also shown promising results. In a mouse model of toxicity-induced acute liver failure, transplantation of mouse differentiated biliary duct organoids derived from Lgr5^+^ stem cells generated detectable organoid-derived nodules in 20-40% of cases (Huch et al. 2013). Although engraftment rate was low (approximately 1%), a significant increase in survival of the grafted group was observed compared to the ungrafted group, indicating that the transplanted cells contributed to liver function repair (Huch et al. 2013). In follow-up studies using the same injury model, the transplantation of mouse (Peng et al. 2018) and human fetal (Hu et al. 2018) hepatocyte organoids, generated much more extensive engraftment indicating that the efficiency of engraftment may be enhanced by transplantation of the most physiologically relevant cell type.

Recently, Xiao and colleagues (Xiao and Deng 2020) generated induced sensory ganglion organoids exhibiting molecular features, subtype diversity, electrophysiological and calcium response properties, and innervation patterns characteristic of peripheral sensory neurons, which may serve as a source for cell replacement therapy. Yoshihara and colleagues (Yoshihara and O'Connor 2020) generated human islet-like organoids (HILOs) from iPSC, which provides a promising alternative to cadaveric and device-dependent therapies in the treatment of diabetes. HILOs contain endocrine-like cell types that, upon transplantation, rapidly re-establish glucose homeostasis in diabetic NOD/SCID mice. Overexpression of the PD-L1 protected HILO xenografts such that they were able to restore glucose homeostasis in immune-competent diabetic mice.

Organoids combined with 3D printing were recently introduced to build the 3D architecture of tissue, which may find widespread applications in regenerative medicine. Firstly, Zhang and his colleagues (Zhang et al. 2016) established the technique that can be transformed into human cardiomyocytes derived from iPSCs to construct endothelialized human myocardium. Then, Creff and colleagues (Creff et al. 2019) provided the possibility of creating artificial 3D scaffolds that match the size and structure of mouse intestinal crypt and villi. Moreover, Homan and colleagues (Homan et al. 2019) built a model that had the ability to induce substantial vascularization and morphogenesis of renal organs *in vitro* under flow conditions opening up a new way for the study of renal development, disease and regeneration.

Though above studies indicate the potential application of organoids in regenerative medicine, many problems need to be solved before organoids are put into clinical use. For example, integration upon transplantation requires optimization, and animal-based 3D ECM matrix used for organoid culture need to be replaced with a synthetic matrix.

### Organoids as a tool for immunotherapy

**Fig. 11:**
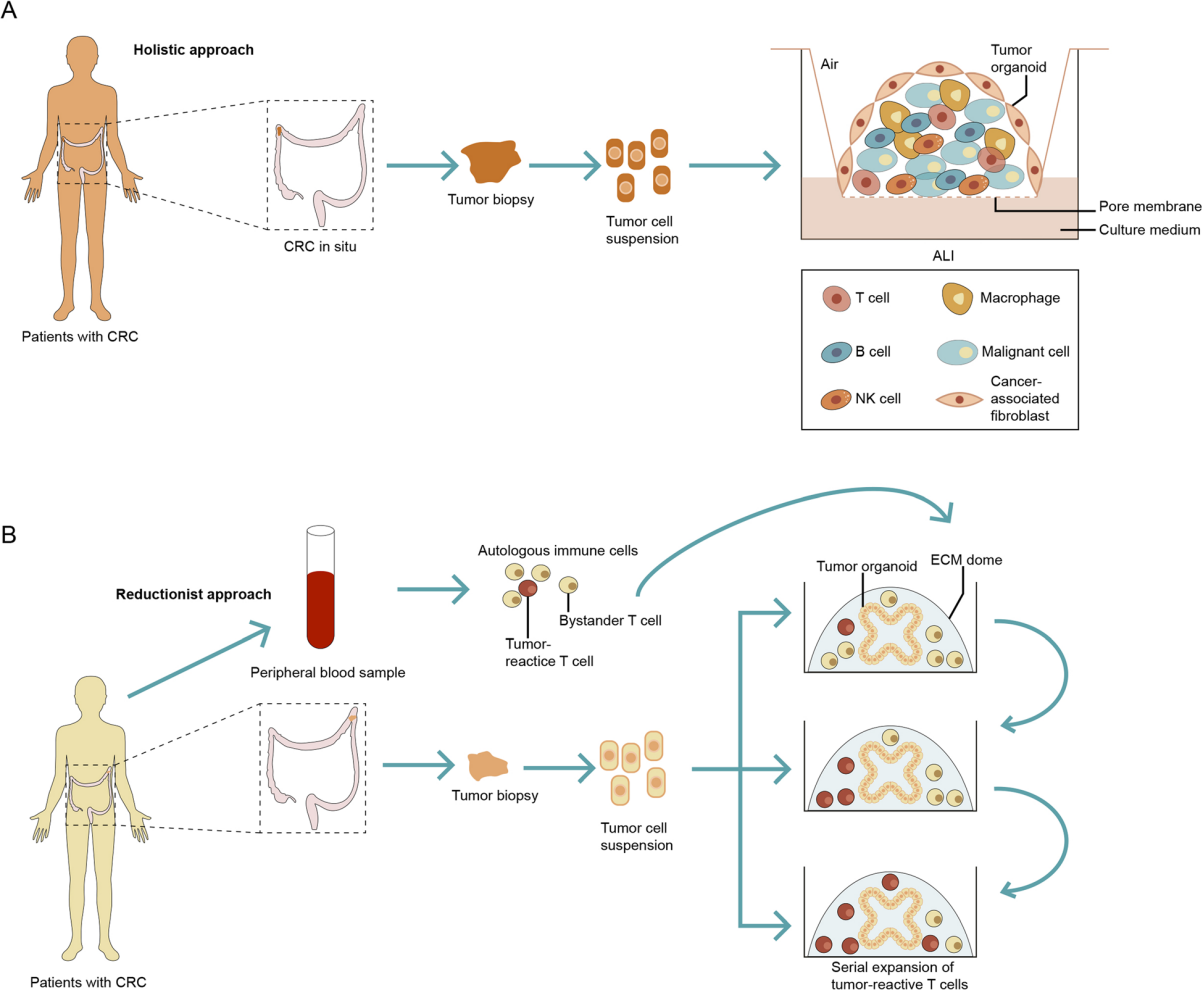
Co-culture systems of organoid and immune cell in immuno-oncology research. Two main methods are currently being adopted: **a** Holistic approach (up). Tumor biopsies are cultured in ALI in the entire tumor microenvironment as a cell suspension of all cell types, including immune cells and other non-epithelial cell types. **b** Reductionist approach (down), epithelial organoids are derived from tumor biopsies and are then co-cultured with autologous immune cells derived from the peripheral blood of the same patient. CRC, colorectal cancer; ALI, air-liquid interphase; ECM, extracellular matrix; NK cell, natural killer cell

The tumor microenvironment consists of various non-epithelial cell types, including immune cells and stromal cells, which greatly affects therapeutic responses. However, it is a major challenge to model tumor microenvironment. Much of our knowledge regarding the tumor microenvironment was studied on cell lines and PDX models. However, cell lines are insufficient to recapitulate the heterogeneity of tumor cells, while the microenvironment of PDX models mainly depend on the mouse immune system, which cannot adequately recapitulate the human immune system. Cancer immunotherapy has emerged as a promising therapeutic developments that take advantage of a patient’s own immune system to eradicate tumor cells (Mellman and Coukos 2011), and several organoid-based models have been established to study immunotherapy response (Fig. 11).

#### Holistic approach based on ALI culture method

Recently, a holistic approach based on the ALI method (mentioned in Section 1.2), which preserved the tumor epithelium and its stromal microenvironment *in vitro,* was described using PDOs of various cancer types, including colorectal and lung cancers (Neal et al. 2018). In addition to stromal fibroblasts, cellular immune components such as tumor-associated macrophages, CTLs (Cytotoxic T lymphocyte), T H cells (T helper cells), B cells, natural killer (NK) cells and natural killer T (NKT) cells were also readily maintained for up to 30 days in the organoid cultures. The organoid cultures also preserved the T cell receptor (TCR) heterogeneity of the T cells found in the parental tumor. The authors used these organoids to model immune checkpoint blockade, leading to the expansion and activation of tumor antigen-specific T cells and subsequent killing of tumor cells.

#### Reductionist approach based on WENR epithelial culture method

Adoptive cell therapy is a promising immunotherapy. In this method, autologous immune cells are expanded *in vitro* followed by the subsequent transplantation of these cells back into the patient, to enhance the immune response against a tumor. Using this strategy, durable regression of melanoma was achieved by *in vitro* expansion of autologous tumor-infiltrating lymphocytes (TILs) (Rosenberg 2015). However, this approach requires resected specimens from which TILs can be obtained. A strategy to avoid resection is to isolate peripheral blood lymphocytes, activate these cells *in vitro* by co-culture with tumor cells. For this strategy, tumor-derived organoids are a highly useful source of tumor cells for co-culture: Tumor organoid cultures can be efficiently established from a small tissue sample through biopsy, and tumor-derived organoids are heterogeneous and recapitulate the genetic and histological characteristics of the parental tumors. Dijkstra and colleagues (Dijkstra et al. 2018) were thus able to obtain tumor-reactive T lymphocytes from peripheral blood lymphocytes after 2 weeks of co-culture with tumor organoids derived from non–small cell lung cancer and MSI-H CRC. Before co-culture, organoids were stimulated with IFN-γ to enhance antigen presentation. PD-1 blocking antibody, IL-2, and anti-CD28 were added to enhance T cell activation. After co-culture, T lymphocytes were activated, as demonstrated by expression of IFN-γ and CD107a. Accordingly, after an additional 3 days of co-culture of activated T lymphocytes with tumor organoids, the survival of the tumor organoids was reduced, while matched normal organoids were unaffected.

Cancers with low mutational burden and stable tumor antigen presentation may be suitable targets for chimeric antigen receptor (CAR)-engineered T cells. Studies on B cell malignancies showed promising results (June 2018). In solid tumors, the therapeutic application of CAR T cells has been hindered by side effects that arise from targeting overexpressed native antigens that are, not exclusively expressed by tumors. Thus, for solid tumors, preclinical models are needed to allow for CAR-mediated cytotoxicity testing. Recently, a luciferase-based quantification assay has been developed to test CAR T cell-mediated cytotoxicity against PDOs (Schnalzger et al. 2019). Instead of using CAR-engineered T cells, the researchers adopted an NK cell line with non-MHC restricted cytolytic activity to target research. The efficiency of the system was confirmed by using CAR-engineered NK-92 cells directed toward *EPCAM*, an epithelial marker overexpressed by cancers. CAR-mediated cytotoxicity was observed against organoids derived from both normal colon tissue and CRC tissue presenting either peptide (Schnalzger et al. 2019). Subsequently, the authors engineered CARs targeting EGFRvIII, a neoantigen that is widely expressed by solid cancers. In a competitive co-culture assay, EGFRvIII-specific CAR NK cells killed EGFRvIII-expressing organoids derived from tumors efficiently but not organoids derived from normal tissue. Finally, the team generated CARs targeting antigens specific to subgroup of CRC that overexpress the WNT ligand receptor FZD upon loss of expression of its antagonists *RNF43*/*ZNRF3*. To test possible side effects of FZD-specific CARs, the authors evaluated cytotoxicity against normal colon organoids as well as different gene-edited organoid lines deficient for both *RNF43* and *ZNRF3* or for *APC*. These co-culture assays illustrated that the cytolytic activity of the FZD-specific CAR NK cells was not specific to the mutated organoid lines, suggesting that such approaches may have marked side effects if used therapeutically. Though effective target has not been found, the platform can be widely used to evaluate CAR efficacy and tumor specificity in a personalized manner.

In summary, co-cultures of cancer organoids and immune cells have become a highly promising strategy for personalized immunotherapy for cancer patients.

## Challenges

**Fig. 12:**
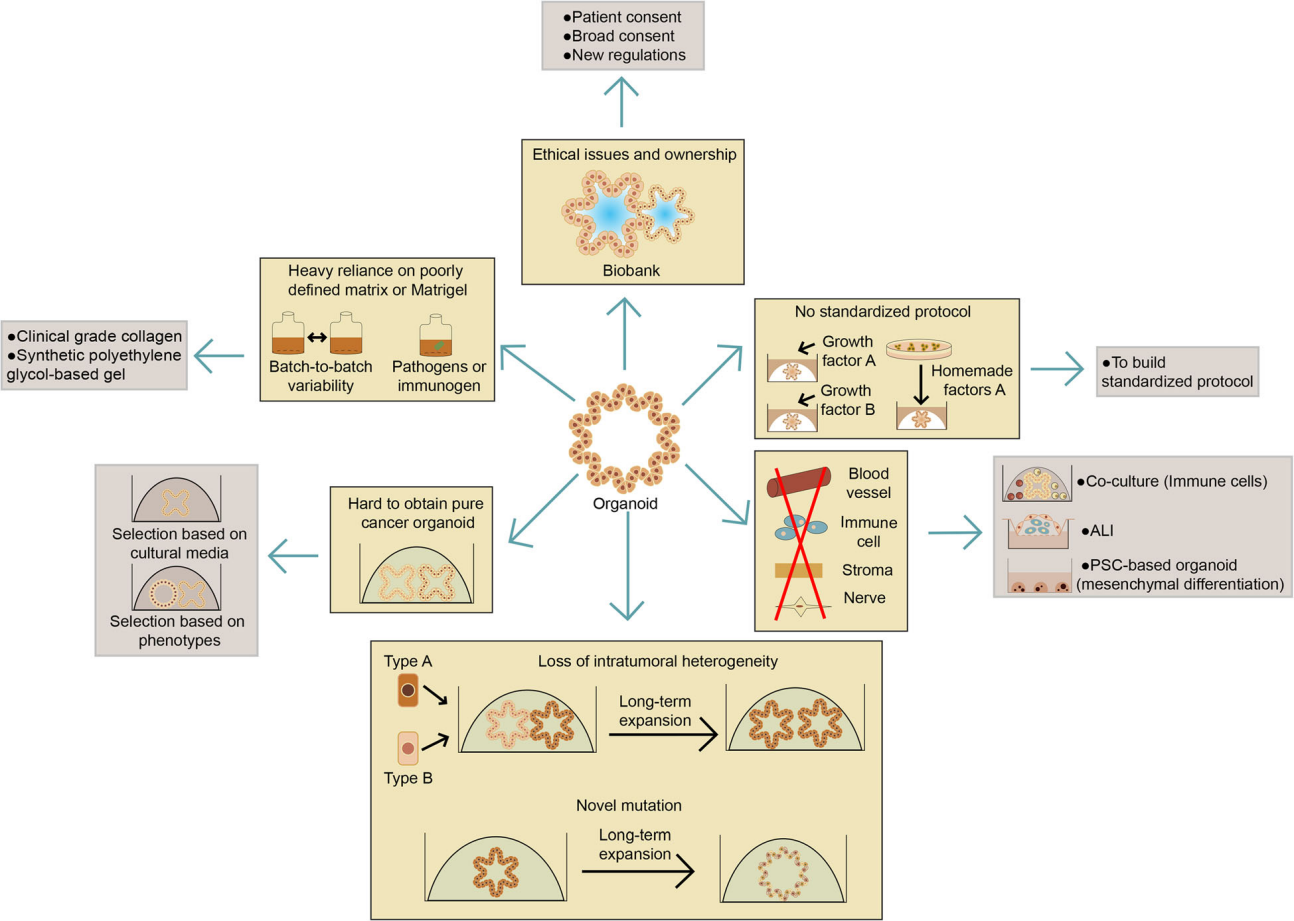
Schematic summarize of current limitations in organoids culture development (yellow) and methods to overcome these issues (brown)

Organoids are robust research tools for the study of human development and disease. However, there are hurdles and limitations associated with using organoids (Fig. 12). First, culture approaches are not standardized. ASCs-derived organoids are established under distinct culture conditions in each laboratory. To reduce the cost, cost-efficient small molecule compounds were used to replace growth factors (Li et al. 2018;Yin et al. 2014). Besides, homemade niche factors produced by various cell lines has been used commonly to culture organoids. However, this trend will result in experimental variation into organoid studies across different research labs.

Second, organoid culture requires the use of Matrigel or other animal-based matrix extract to enable cells to aggregate into 3D structures. These extracts suffer from batch-to-batch variability in their composition, which may affect the reproducibility of experiments. In addition, they may carry unknown pathogens and are potentially immunogenic when transplanted to humans, limiting the application of organoids in a clinical transplantation setting. This may be solved by culturing with clinical grade collagen, which has been successfully used for colon organoids culture and expansion (Yui et al. 2012). Steps toward fully defined culture conditions have been made with the development of a synthetic polyethylene glycol-based gel that sustains the short-term growth of mouse ASC-derived intestinal organoids (Gjorevski et al. 2016). However, this matrix remains to be optimized for the long-term expansion of intestinal organoids and f non-intestinal organoids.

Third, obtaining pure tumor organoids is another critical problem for researchers. Many studies have reported that tumor organoids can be overgrown and contaminated by matched normal organoids. One of the most widely used methods to acquire pure tumor organoids is to select tumor cells that harbor the most common mutations in its cancer type. For example, tumor organoids derived from CRC can be selectively upon withdrawal of Wnt3a and R-Spondin1. However, this method is not applicable to all cancers, and some specific cancer subtypes. More importantly, intra-tumoral heterogeneity would inevitably be lost upon selection. Some labs have suggested obtaining pure tumor organoids based on growth phenotypes. However, not all tumor organoids have clear morphological differences to normal organoids. More researches are needed to establish appropriate approaches for obtaining pure tumor organoids.

Fourth, for ASCs-derived organoids, only the epithelial compartment of organs is represented, while blood vessels, immune cells, stroma, and nerves are lacking. As mentioned above, many groups have focused co-culturing organoids with various types of cells, analogous to what has already been done with immune cells (Dijkstra et al. 2018), or adopted unconventional organoid culture methods, such as the ALI method (Neal et al. 2018;Ootani et al. 2009). Besides, several PSC-based organoids are able to undergo mesenchymal differentiation to generate subepithelial myofibroblasts and smooth muscle (Spence et al. 2011).

Last, the development of a model based on living human tissues that can be stored and expanded in biobanks-potentially forever-has raised a set of ethical issues regarding informed consent and ownership (Bredenoord et al. 2017). For organoid biobanks, patient consent is required. The most common type of patient consent restricts the use of a patient’s material to only a specific research aim. However, biobanks are useful for researchers in multiple fields, and the use of biobanks in a combination of fields may provide potentially synergistic data. To solve this problem, Bredenoord and colleagues (Boers and van Delden 2015;Bredenoord et al. 2017) have suggested that a broad consent be used for governance. This broad consent would allow donors to make informed decisions about how their samples are used after they have been provided with relevant information about the establishment and regulation of the biobank. Another issue that has arisen with the development of living biobanks is ownership. Organoids are increasingly used by commercial parties as tools for drug development or in validation studies. Such uses will inevitably result in patentable compounds. It may be helpful to include regulations covering the distribution of any financial gains from intellectual property among stakeholders in the governance of the biobanks.

Furthermore, in terms of the culture conditions of tumor organoids, intra-tumoral heterogeneity could be lost during passages for that the culture media might not favor the growth of all tumor subclones equally effectively. Additionally, novel mutations would be acquired during long-term expansion. Collectively, these drawbacks deserve further attention, and more work is needed to improve organoids culture technologies. Despite these limitations, organoid technology holds great promise as a robust tool for basic, translational and clinical research for human development and disease modeling.

## Outlooks

In this review, we have demonstrated the potential of organoid technology for modeling genetic, infectious and malignant disease, as well as for drug development and personalized medicine. The basic and translational applications of organoids are expected to expand in the future.

Regarding epithelial genetics, organoids have potential especially for rare diseases due to their high expansion ability and genetic stability. In infectious diseases, the mechanisms of virus-induced malignant transformation, for example, by Epsteini-Barr virus in gastric and nasopharynx cancers, may be studied in long-term co-cultures of the virus with normal epithelium from the respective organ. In the studies of malignancies, culture conditions need to be developed for many types of carcinomas and for sarcomas and melanomas.

The optimization of drug screening will be a pivotal use of organoids in personalized medicine by adopting biobanks of different cancers. This enables many compounds to be screened for a specific disease or a specific compound to be screened for many forms of a given disease. Moreover, multiple organoids can be derived from the same cancer patient over time to assess drug response and developing resistance to targeted drugs and to predict patient outcome.

In the area of regenerative medicine, there is still a long way to go for the transplantation of organoids as therapy. Several hurdles, including the development of a non-animal-based alternative for Matrigel and efficient delivery procedures, remain to be overcome.

In conclusion, the worldwide application of organoids has contributed to unprecedented advances in research on human development and diseases. Even though current organoid systems show some limitations and require further optimization for use in disease modeling and personalized medicine, they will continue to be valuable tools in basic and translational research.

## Abbreviations

2D: Two-dimensional
3D: Three-dimensional
PDX: Patient-derived xenograft
PSCs: Pluripotent stem cells
iPSCs: induced Pluripotent stem cells
ESCs: Embryonic stem cells
ASCs: Adult stem cells
EHS: Engelbreth Holm Swarm
EGF: Epidermal growth factor
BMP: Bone morphogenetic protein
ECM: Extracellular matrix
PDOs: Eatient-derived organoids
WENR method: Wnt3a+EGF+Noggin+R-spondin-1
NACE: N-acetylcysteine
NICO: Nicotinamide
RSPO: R-spondin-1
PGE2: Prostaglandin E2
DHT: Dihydrotestosterone
CRC: Colorectal cancer
ALI: Air-liquid interface
TILs: Tumor infiltrating lymphocytes
TA: Transit-amplifying
WGS: Whole-genome sequencing
CRISPR: Clustered regularly interspaced short palindromic repeats
Cas9: CRISPR associated protein 9
NHEJ: non-Homologous end joining
HDR: Homology-directed repair
CF: Cystic fibrosis
CFTR: CF transmembrane conductance regulator
cAMP: cyclic Adenosine monophosphate
FSK: Forskolin
CLC: Cholangiocyte-like cells
PDECs: Pancreatic ductal epithelial cells
MVIX: Microvillus inclusion disease
*STX3*: *Syntaxin-3*
A1AT: α1-antitrypsin
AGS: Aicardi-Goutières syndrome
ZIKV: *Zika virus*
WHO: World Health Organization
TLR3: Toll-like receptor 3
JEV: *Japanese encephalitis virus*
JE: Japanese encephalitis
HBV: *Hepatitis B virus*
*HuNoV*: *Human Norovirus*
HBGA: Histo-blood group antigen
RSV: Respiratory syncytial virus
EV71: *Enterovirus 71*
*S. typhi*: *Salmonella typhi*
*C. difficile*: *Clostridium difficile*
CDI: *C. difficile* infection
TcdA: Toxin A
TcdB: Toxin B
*H. pylori*: *Helicobacter pylori*
CIN: Chromosomal instability
HIV: Human immunodeficiency virus
shRNA: Short hairpin RNA
SSAs: Sessile serrated adenomas
MLH1: MutL homolog 1
MSI: Microsatellite instability
PDAC: Pancreatic ductal adenocarcinoma
SRCC: Signet-ring cell carcinoma
LSL: Lox-stop-lox
PanINs: Pancreatic intraepithelial neoplasias
KCTS: *KRAS*^*G12V*^, *CDKN2A*, *TP53* and *SMAD4*
PTRN: *P53, PTEN, RB1 and NF1*
AR: Androgen receptor
*TMPRSS2*: Transmembrane protease serine 2
ETS: E26 transformation-specific
ERG: ETS-related gene
TRGs: Tumor regression scores
AUC: Area under the curve
IPMN: Intraductal papillary mucinous neoplasm
ER: Estrogen receptor
PR: Progesterone receptor
HGSC: High-grade serous ovarian cancer
LGSC: Low-grade serous ovarian cancer
PARP: Poly (ADP-ribose) polymerase
HNSCC: Head and neck squamous cell carcinoma
CAR-T: Chimeric antigen receptor T
NK cell: Natural killer cell
NKT cell: Natural killer T cell
TCR: T cell receptor
TILs: Tumor-infiltrating lymphocytes
CAR: Chimeric antigen receptor
DSS: Dextran Sulfate Sodium
IFN-γ: Interferon-gamma
MHC: Major histocompatibility complex
FZD: Frizzled
NHE3: Sodium/Hydrogen Exchanger 3
MUC2: Mucoprotein2
AKT: Protein kinase B
MAPK: Mitogen-activated protein kinase
EIF3E: Eukaryotic Translation Initiation Factor 3 Subunit E
JAK2: Janus Kinase 2
EGFR: Epidermal growth factor receptor
PRMT5: Protein Arginine Methyltransferase 5
MTAP: Methylthioadenosine phosphorylase
ERK: Extracellular regulated protein kinases
AR: Androgen receptor
DSS: Dextran Sulfate Sodium Salt
CTLs: Cytotoxic T lymphocyte
T H cells: T helper cells
COVID-19: Coronavirus disease-19
SARS-CoV-2: Severe acute respiratory syndrome-coronavirus 2
MGE: Medial ganglionic eminence
hrsACE2: human recombinant soluble ACE2
IWP-2: Inhibitor of Wnt Production 2
VPA: Valproic acid
CHIR: Chicken immunoglobulin-like receptor
DAPT: Dual antiplatelet therapy
SgRNA: Single-guide RNA
FOXG1: Forkhead box G1
HILOs: Human islet-like organoids
